# Synthesis and anticancer activity of thiosubstituted purines

**DOI:** 10.1007/s00044-015-1364-2

**Published:** 2015-03-25

**Authors:** Alicja Kowalska, Małgorzata Latocha, Krystian Pluta

**Affiliations:** 1 Department of Organic Chemistry, School of Pharmacy with the Division of Laboratory Medicine, The Medical University of Silesia, Jagiellońska 4, 41-200 Sosnowiec, Poland; 2 Department of Cell Biology, School of Pharmacy with the Division of Laboratory Medicine, The Medical University of Silesia, Jedności 8, 41-200 Sosnowiec, Poland

**Keywords:** Azathioprine analogs, Dialkylaminoalkylthiopurines, Purinesulfenamides, Purinesulfonamides, Anticancer activity

## Abstract

New thiopurines with the propargylthio, pyrrolidinobutynylthio, sulfenamide, and sulfonamide groups in the pyrimidine ring were synthesized. The anticancer activity of these compounds and previously obtained 2- or 6-substituted azathioprine analogs and dialkylaminoalkylthiopurines were tested in vitro against three cell lines: glioblastoma SNB-19, melanoma C-32, and human ductal breast epithelial tumor T47D. 2-Chloro-7-methyl-6-pyrrolidinobutynylthiopurine (**5b**) was the most potent compound against SBN-19 and C-32 cell lines with the activity similar to cisplatin (EC_50_ = 5.00 and 7.58 μg/ml, respectively). The dialkylaminoalkylthio derivatives (**4b**, **4c**, **4e**, and **4f**) showed good activity against SBN-19 cell line (EC_50_ < 10 μg/ml). The azathioprine analogs (**2a**, **2b**, and **3a**) were more active than azathioprine against SBN-19 and C-32 cell lines. The sulfenamide and sulfonamide derivatives of purine were very weak active against tested cell lines. All studied thiopurines were less toxic than cisplatin.

**Electronic supplementary material:**

The online version of this article (doi:10.1007/s00044-015-1364-2) contains supplementary material, which is available to authorized users.

## Introduction

The sulfur-containing purines: 6-mercaptopurine, 6-thioguanine, and azathioprine, have been considered as important and effective drugs used in cancer chemotherapy, for immunosuppression in kidney or heart transplantation and autoimmune diseases (Steurer *et al*., 2006; Hawwa *et al*., 2008; Relling *et al*., 2013; Rao *et al*., 2013). 6-Mercaptopurine is widely used as an antileukemic agent in the lymphoproliferative disorders, including lymphoma, childhood acute lymphoblastic leukemia, and other neoplastic conditions (Hawwa *et al*., 2008; Prima *et al*., 2013; Miron *et al*., 2009). Azathioprine, but also 6-mercaptopurine exert immunosuppressive effects and are the treatments of choice for severe chronic inflammatory diseases such as rheumatoid arthritis, ulcerative colitis, Crohn’s disease, inflammatory bowel diseases, systemic lupus erythematosus, and multiple sclerosis (Aldinucci *et al*., 2010; Colombel *et al*., 2010; Daehn and Karran, 2009). Various pharmacological effects of synthetic thiopurines including antiviral, antibacterial, antitumor, and antifungal activity (Lech-Maranda *et al*., 2006; Rao *et al*., 2013) were developed for treatment of patients with infections of human immunodeficiency virus (HIV) and infections caused by herpes and hepatitis virus (Rao *et al*., 2013; Schow *et al*., 1997; Chang-Hyun *et al*., 2001).

However, the use of thiopurines is limited by their toxicities, which include hepatotoxicity, myelosuppression, pancreatitis, and allergic reactions (Prima *et al*., 2013). For this reason, many thiopurine derivatives and analogs have been synthesized for evaluation of their biological activities and reduced toxicity. Recently, 9-substituted derivatives of 6-(t-butoxycarbamylaminohexyl)thiopurine were obtained which showed moderate antibacterial activity (Rao *et al*., 2013); a series of 6-mercaptopurine analogs with 1,2,3-triazole or steroid rings exhibited promising antimalarial and antileishmanial activities (Corrales *et al*., 2011); and biologically active 6-allyldithiopurine and its riboside inhibited cell proliferation and induced apoptosis (Miron *et al*., 2009).

Heterocyclic sulfonamides are used as carbonic anhydrase inhibitors, antibacterial, anti-inflammatory, analgesic, hypoglycemic, antifungal, and antiviral agent (Joseph *et al*., 2010; Guzel *et al*., 2009; Azab *et al*., 2013; El-Sayed *et al*., 2011; Veisi *et al*., 2011). It is known that aryl and heteroaryl sulfonamides may act as antitumor agents through perturbation of cell cycle in the G_1_ phase, distribution of microtubule assembly, or angiogenesis inhibition. They reverse the effect of tumor acidification, consequently inhibit the growth of cancer cell, and suppress tumor invasion mediated by the carbonic anhydrase active side (Zn^2+^) and so inhibit the catalytic ability of this enzyme (El-Sayed *et al*., 2011). Indisulam (E7070) is a novel sulfonamide anticancer agent currently in phase II clinical development for the treatment of several types of cancer. It was shown to act as a nanomolar inhibitor of carbonic anhydrase isoform IX (Sławiński *et al*., 2013; Mirian *et al*., 2011).

The sulfamoyl group has been extensively utilized as an activity-modifying substituent in many different classes of drugs. The synthesis of 2-substituted 7- or 9-methylpurines containing a sulfanilamide group at the positions 2, 6, or 8 of the purine ring with moderate antibacterial activity was reported by the reaction of chloropurines with sodium sulfanilamide (Beaman *et al*., 1966). Structural modification of the amino group at C-6 atom of adenosine leads to selective A_1_ receptor agonists, while modification at C-2 atom confers high potency and selectivity on A_2A_ receptors. The synthesis of N-9- and 6-amino monosulfonyladenine and disulfonyladenine derivatives and its sulfonylated nucleoside with high potency and selectivity on A_1_ and A_2A_ receptor agonists has been described (Zinic *et al*., 2001). A number of 6-sulfenamide, sulfinamide, and sulfonamide derivatives of 6-mercaptopurine, 6-thioguanine, and related ribonucleosides have been synthesized and evaluated for antileukemic activity in mice (Revankar *et al*., 1990). It is quite interesting that a subtle change to an oxidized sulfur atom in the form of sulfonamide resulted in a new group of purine derivatives possessing significant antitumor properties. Majority of the tested compounds exhibited very significant anti-L1210 cell activity (Revankar *et al*., 1990; Robins *et al*., 1991).

Oligonucleotides containing 1-alkynyl-6-thiopurine or 6-thioguanine bases demonstrate potent antiviral activity in several assays, including the human immunodeficiency virus reverse transcriptase enzyme assay (Broom *et al*., 1997). The purine derivatives possessing the alkynyl groups at position 6 are cytokinin analogs with profound plant growth-stimulating effect and have attracted considerable interest as potential anticancer compounds. All 6-phenylacetylenes and certain enyne of the purine are highly toxic against K-562 cells and exhibit cytotoxic activity against a human chronic myelogenous leukemia cell line comparable to well-known anticancer drugs (6-mercaptopurine and fludarabine) (Brathe *et al*., 2003). Many adenine and adenosine derivatives with 2-, 6-, or 8-alkynyl substituents are selective adenosine subtypes A_1_, A_2A_, A_2B_, and A_3_ receptor antagonists. 2-Alkynyl-8-aryl adenine derivatives bear methyl or an amide moiety at the position 9, as A_2B_ receptor antagonists have hypoglycemic activity and may be used as antidiabetic agents (Harada *et al*., 2001). Stimulating A_2A_ receptor by 2-adenosine propargyl phenyl ether composition resulted in vasodilating activity and potential of using in coronary artery disease (Zablocki *et al*., 2001). Introduction of alkynyl chains on C-8 atom of adenosine led to very selective antagonists of the A_3_ receptor (Volpini *et al*., 2001). Some 2-alkynyl and 2,(6-amino)dialkynyl adenosine derivatives show high affinity and different degrees of selectivity for A1, A_2A_, and A3 receptor (Volpini *et al*., 2001).

Our previous research demonstrated the effective synthesis of thiosubstituted purines such as 2- or 6-substituted azathioprine analogs (Kowalska and Pluta, 2008; Kowalska *et al*., 2009) and dialkylaminoalkylthiopurines (Kowalska and Pluta, 2012). The aim of this study was to elaborate synthesis of new thiopurines with the propargylthio, pyrrolidinobutynylthio, sulfenamide, and sulfonamide groups in the pyrimidine ring, to examine anticancer activity of not only those compounds but also previously synthesized thiopurines and to discuss the structure–activity relationship.

## Result and discussion

### Chemistry

**Scheme 1:**
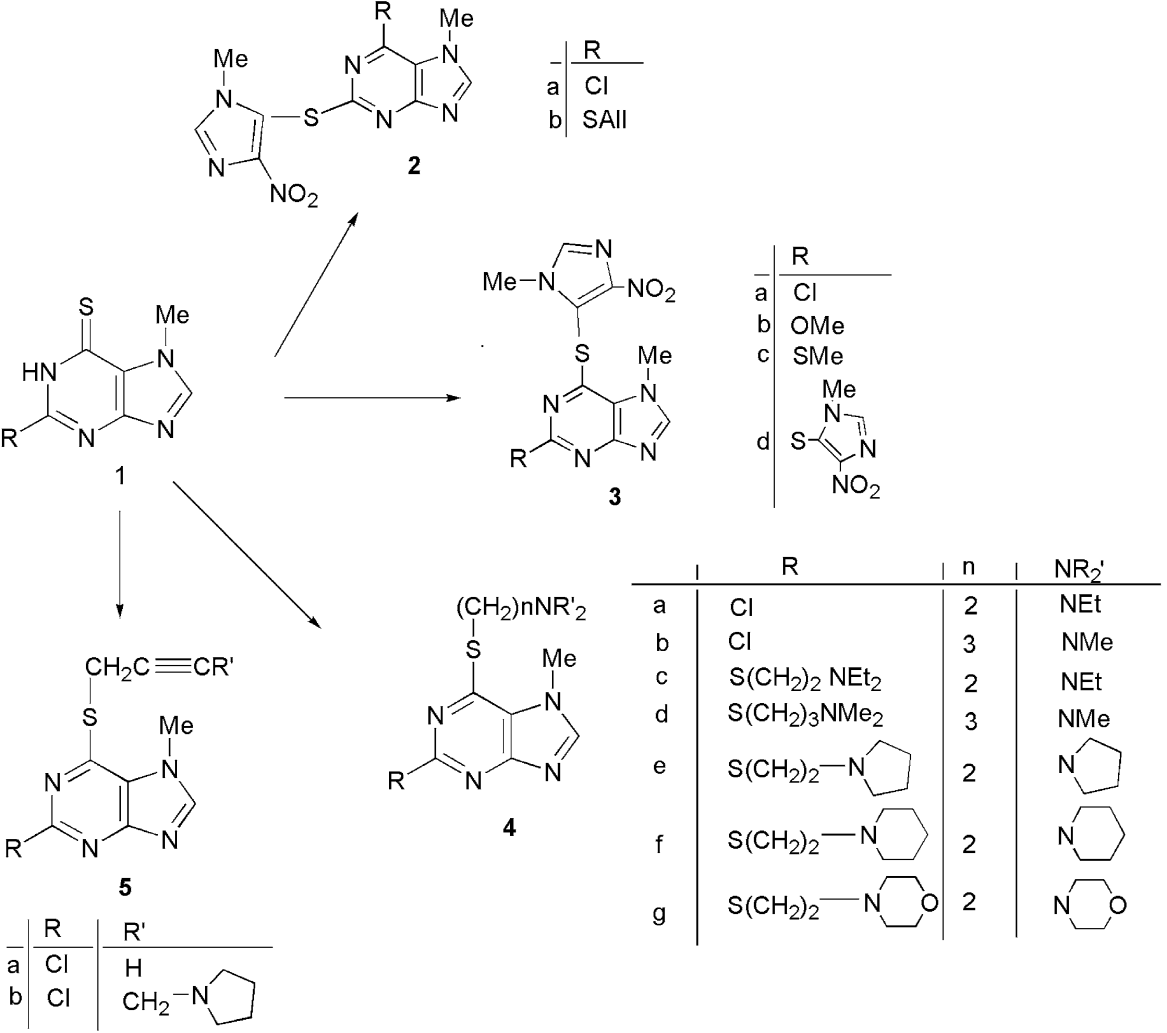
Synthesis of 2- or 6-substituted azathioprine analogs **2**, **3**, dialkylaminoalkylthiopurines **4**, propargylthio- and pyrrolidinobutynylthiopurines **5**

All thiosubstituted 7-methylpurines were obtained from 2-substituted 7-methylpurine-6-thiones **1a–c** and 7-methylpurine-2,6-dithiones **1d**. Purinyl sulfides **2–4** with the pharmacophoric 1-methyl-4-nitroimidazol-5-yl and dialkylaminoalkyl groups in positions 2 and 6 were obtained according to the procedures published previously (Kowalska and Pluta, 2008; Kowalska *et al*., 2009; Kowalska and Pluta, 2012). The alkynylthio derivatives **5** were obtained from 2-chloro derivative **1a** and propargyl bromide and further via Mannich reaction with pyrrolidine (Scheme 1).

The traditional and general method for preparing sulfonamides is based on reaction of sulfonyl chloride with ammonia or amines. The aryl sulfonyl chlorides may be obtained from thiols, sulfides, thioacetals, or thiocarbamines by oxidative chlorination with chlorine gas in aqueous acid (Veisi *et al*., 2011; Mirian *et al*., 2011; Ashfaq *et al*., 2013; Narasaiah *et al*., 2012; Rehman *et al*., 2012). 2-Chloro-7-methyl-6-purinethione **1a** was chosen as a model compound to prepare S-benzylthiopurine **6**. It is known that azinesulfonyl chlorides with the chlorosulfonyl group in aza-activated positions readily lose sulfur dioxide to form chloroazines (Beaman and Robins, 1961; Beaman, 1963; Revankar *et al*., 1990).

**Scheme 2:**
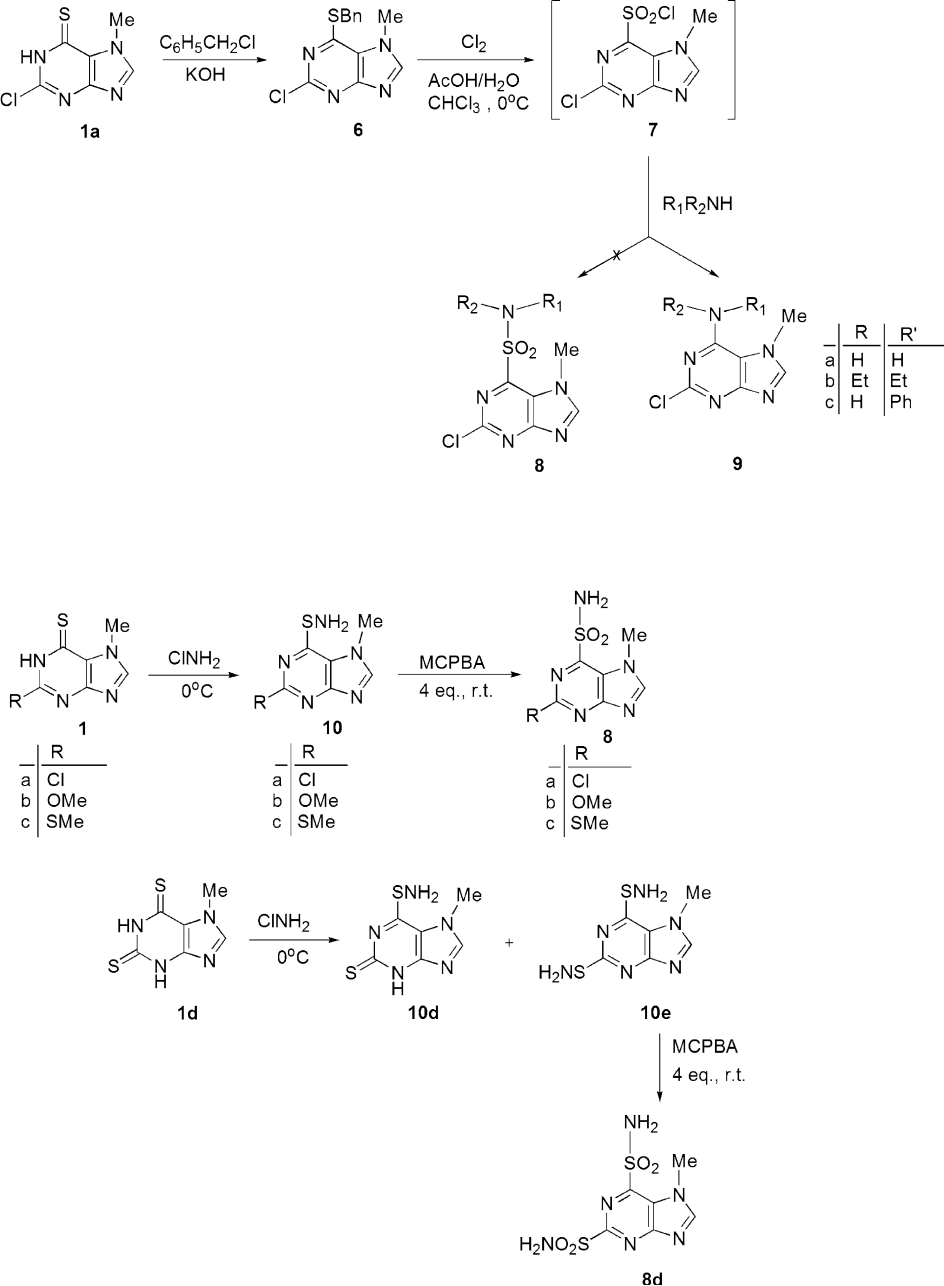
Synthesis of amine **9**, sulfenamide **10**, and sulfonamide **8** derivatives of purines

Two-step synthesis of purinesulfonamide **8** via oxidation and chlorination of sulfide **6** with chlorine in acetic acid and subsequent transformation of sulfonyl chloride **7** with ammonia, diethylamine, or aniline failed. In this case, the highly instable chlorosulfonyl group lost sulfur dioxide, and resulted chloropurine reacted with ammonia and amines to give 2-chloro-6-amine derivatives **9a–c** with 71–86 % yield (Scheme 2).

Therefore, the sulfonamide compounds were obtained from purinethione via amination and oxidation. The procedure of S-amination of azinethiones with chloramine solution has been widely used in the preparation of purine-, pyridine-, pyrimidine-, and benzothiazolesulfenamides (Revankar *et al*., 1990; Robins *et al*., 1991; Hurley and Robinson, 1965; Schoenwald *et al*., 1984). For 2-Substituted 7-methyl-6-purinethiones **1a–c**, S-amination with a chloramine solution at 0 °C to sulfenamide **10a–c** in 85–92.5 % yield was carried out. In the case of purinedithione **1d**, two products were obtained: disubstituted compound as the main product, purine-2,6-disulfenamide **10e** (66 % yield) and purine-6-sulfenamide **10d** (17.5 % yield). Treatment of sulfenamides **10** with 4 molar equivalent of 3-chloroperoxybenzoic acid (MCPBA) at room temperature in ethanol gave purinesulfonamides **8a–d** in 66–79 % yield. Lesser equivalents of MCPBA led to sulfenamides (Revankar *et al*., 1990).

**Scheme 3:**
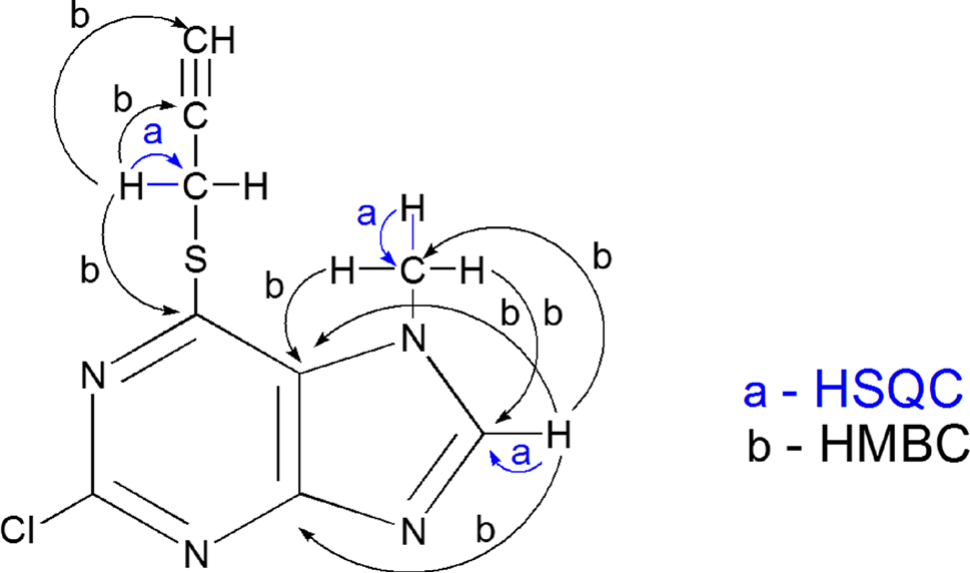
2D NMR experiments for compound **5a**

The structure of all new compounds was determined by the spectral data analysis: ^1^H and ^13^C NMR, EI, CI or FAB MS, and HR MS. ^1^H NMR spectra of new compounds revealed H-8, NCH_3_ proton signals, and protons from C-2 (OCH_3_, SCH_3_) and C-6 (CH_2_C_6_H_5_, NH_2_, N(C_2_H_5_)_2_, NHC_6_H_5_, SCH_2_CCH, SCH_2_CCCH_2_NC_4_H_8_, SNH_2_, SO_2_NH_2_) substituents. ^13^C NMR spectra showed primary, secondary, tertiary, and quaternary carbon signals. In order to assign all of these signals, 2D NMR techniques (HSQC—the primary, secondary, and tertiary carbon atoms connected with the hydrogen atoms and HMBC—the primary, tertiary, and quaternary carbon atoms connected with the hydrogen atoms via two and mainly three bonds) for selected compounds (**5a**, **10e**) were used. For compounds **5a**, in the HSQC spectrum the H-8 proton at 8.59 ppm correlated with the signal at 151.09 ppm (C-8), the SCH_2_ proton at 4.22 ppm correlated with the signal at 18.09 ppm (C, SCH_2_), and the proton at 4.04 ppm (NCH_3_) correlated with the signal at 34.84 ppm (C, NCH_3_). The HMBC spectrum revealed correlation via three bonds between the H-8 proton at 8.59 ppm and the carbon signals at 34.84 ppm (C, NCH_3_), at 123.16 ppm (C-5), and at 160.52 ppm (C-4). The proton signal at 4.22 ppm (H, SCH_2_) correlated with the signals of carbon atoms via two bonds at 79.55 ppm (C, CCH) and via three bonds at 74.67 ppm (C, CH) and at 153.99 ppm (C-6). The proton signal at 4.04 ppm (H, NCH_3_) correlated via three bonds with the signals at 123.16 ppm (C-5) and at 151.09 ppm (C-8). The proton–carbon correlation was presented in Scheme 3.

### Anticancer activity

For the biological tests, 20 thiopurines of five types were selected:

- the azathioprine analogs containing the methylnitroimidazolylthio group in position 2 (compounds **2a**, **2b**), in position 6 (**3a–c**), and in both positions (**3d**),
- the dialkylaminoalkylthio derivatives containing that substituent with an open-chain amine group in positions 6 (**4a, 4b**), 2 and 6 (**4c**, **4d**) and with a cyclic amine group (pyrrolidine, piperidine, and morpholine) in positions 2 and 6 (**4e–g**),
- the alkynylthio derivatives containing the propynylthio (**5a**) and pyrrolidinobutynylthio (**5b**) groups,
- the sulfenamide derivative (**10a**),
- the sulfonamide derivatives with that group in positions 6 (**8a–c**), and 2 and 6 (**8d**).

The anticancer activity of the obtained compounds was investigated in vitro using cultured glioblastoma SNB-19, melanoma C-32, and human ductal breast epithelial tumor T47D cell lines. Normal human fibroblasts (HFF-1) were used as a control, and azathioprine and cisplatin as reference drugs. Table 1 contains the activity of thiopurines as the EC_50_ values.

**Table 1:** Antiproliferative activity of thiopurines

Compound	Antiproliferative activity EC_50_ (μg/ml)			
	SBN-19	C-32	T47-D	HFF-1
**2a**	17.92	21.77	41.11	>50
**2b**	33.26	35.33	49.60	>50
**3a**	30.38	12.79	>50	34.93
**3b**	>50	>50	>50	>50
**3c**	>50	>50	48.63	>50
**3d**	>50	>50	>50	>50
**4a**	28.77	22.47	41.27	>50
**4b**	8.45	>50	>50	>50
**4c**	9.82	33.55	24.00	40.00
**4d**	36.28	>50	>50	>50
**4e**	7.95	27.05	38.40	35.41
**4f**	9.18	21.80	32.25	45.50
**4g**	>50	>50	>50	>50
**5a**	30.95	35.35	44.30	48.95
**5b**	5.00	7.58	46.00	43.12
**8a**	>50	>50	>50	>50
**8b**	35.02	>50	>50	>50
**8c**	>50	>50	>50	>50
**8d**	>50	>50	>50	>50
**10a**	>50	>50	>50	>50
Azathioprine	37.20	35.62	39.00	55.88
Cisplatin	4.45	6.28	8.79	30.00

The most active was 2-chloro-7-methyl-6-pyrrolidinobutynylthiopurine (**5b**) with the EC_50_ values of 5.00 and 7.58 μg/ml against SNB-19 and C-32 cell lines, comparable to the cisplatin effect. This compound exhibited good selectivity and low toxicity, being weak active against T47D cell line and normal fibroblasts HFF-1. The introduction of the pyrrolidine ring to the alkynyl chain enhanced the activity (compound **5b** vs. **5a**).

Good activity against SNB-19 cell line was observed for the dialkylaminoalkylthio derivatives (**4b**, **4c**, **4e**, and **4f**) with the EC_50_ values below 10 μg/ml. The compounds with the ethyl chain were more active than those with the propyl chain. The most active was compound **4e** with the two pyrrolidinoethylthio groups. The activity against other cell lines was lesser as well as their toxicity.

The azathioprine analogs with the imidazolylthio group in position 2 were more active than those with that group in position 6 with an exception of compound (**3a**) which exhibited good activity against C-32 cell line. The most active azathioprine analogs (**2a**, **3a**) possessed the chlorine atom in position 2 or 6. Both compounds were more active than azathioprine (against first two cell lines) and compounds containing the methoxyl and thioalkyl groups (**2b**, **3b**, **3c**). Compound **3d** with two imidazolylthio groups turned out to be unexpectedly inactive. In our opinion, such an effect was the result of the unusual spatial arrangement of those groups directed to N-1 atom with the donor–acceptor interaction between the imidazole rings, as it was observed in X-ray analysis of the monocrystal **3d** (Kowalska *et al*., 2009).

The sulfenamide and sulfonamide derivatives are very weak active or inactive. The glioblastoma SNB-19 was most sensitive and breast tumor T47D least sensitive cell line for thiopurines. All studied compounds were less toxic than cisplatin.

### Conclusion

In search for novel thiopurine derivatives, we described synthesis of 2-chloro-6-alkynylthio-7-methylpurines **5a**, **5b** from 2-chloro derivative and propargyl bromide and further via Mannich reaction with pyrrolidine. Other types of thiopurines bearing the sulfenamide **10a–e** and sulfonamide **8a–d** groups were obtained through S-amination of purinethiones **1a–d** and oxidation with 3-chloroperoxybenzoic acid.

New thiopurines **5**, **8**, and **10**, previously synthesized azathioprine analogs **2** and **3**, and dialkylaminoalkylthiopurines **4** were investigated as antitumor agents. The most potent compound against SBN-19 and C-32 cell lines was 2-chloro-7-methyl-6-pyrrolidinobutynylthiopurine **5b** with the activity similar to cisplatin. The dialkylaminoalkylthio derivatives **4b**, **4c**, **4e**, and **4f** showed good activity against SBN-19 cell line. The azathioprine analogs **2a**, **2b**, and **3a** were more active than azathioprine against SBN-19 and C-32 cell lines. The sulfenamide and sulfonamide derivatives of thiopurine were very weak active against tested cell lines. All studied thiopurines were less toxic than cisplatin.

## Experimental

6-Substituted derivatives of 7-methyl-2-(1-methyl-4-nitroimidazol-5-ylthio)purines **2a–b** were prepared from 7-methyl-2,6-di(1-methyl-4-nitroimidazol-5-ylthio)purine **3d** exploiting different reactivity of the imidazolylthio groups toward nucleophilic reagents (Kowalska *et al*., 2009). 2-Substituted derivatives of 7-methyl-6-(1-methyl-4-nitroimidazol-5-ylthio)purines **3a–d** were obtained in the reaction of 6-purinethiones with 5-chloro-1-methyl-4-nitroimidazole in 70 % ethanol (Kowalska and Pluta, 2008). 2-Substituted 6-(dialkylaminoalkylthio) **4a–b** and 2,6-bis(dialkylaminoalkylthio) derivatives **4c**–**g** were obtained via the direct S-dialkylamination of the appropriate purinethiones (Kowalska and Pluta, 2012). All commercially available organic solvents and reagents were from Sigma-Aldrich and Chempur and were used without further purification.

The melting points were determined in open capillary tubes on a Boetius melting point apparatus and were uncorrected. ^1^H NMR spectra were recorded on a Bruker AVANS 300 spectrometer operating at 300 MHz and 75 MHz for ^1^H and ^13^C nuclei, respectively, in deuterochloroform and dimethyl sulphoxide-*d*_6_ with tetramethylsilane as internal standard. Shifts were given in ppm, coupling constant (J) values were presented in hertz (Hz), and the abbreviations were as follows: s (singlet), d (doublet), t (triplet), and m (multiplet). Electron impact (EI MS), chemical ionization (CI MS), fast atom bombardment (FAB MS), and high-resolution (HR MS) (in the *m*-nitrobenzyl alcohol and glycerol matrix) mass spectra were run on a Finnigan MAT 95 spectrometer at 70 eV. The reactions were monitored by thin-layer chromatography (TLC) using aluminum sheets coated with silica gel 60_F254_ (Merck) and chloroform–ethanol (9:1) as the solvents. Purity of the synthesized compounds was confirmed by TLC in the same way. Spots were detected by their absorption under UV light (*λ* = 254 nm), and the chromatograms were further visualized by iodine vapor. Column chromatography separations were carried out with Merck Kieselgel 60 or aluminum oxide 90 (Merck) using a mixture of chloroform–ethanol (99:1, v/v) as an eluent.

### *Synthesis of 2*-*chloro*-*6*-*(prop*-*2*-*ynylthio)*-*7*-*methylpurine****5a***

Propargyl bromide (0.17 g, 1.44 mmol) was added to a solution generated from the reaction of 2-chloro-7-methyl-6-thiopurine (0.2 g, 1 mmol) with t-BuOK (0.16 g, 1.44 mmol) in 10 ml of DMF at room temperature for 0.5 h. The reaction mixture was stirred for an additional 24 h at room temperature and then added to 25 ml of water. The resulted solid was filtered off and washed with water to give compound **5a**. It was obtained as a pale yellow solid (0.22 g, 92 %); mp 183–184 °C (EtOH); ^1^H NMR (DMSO-d_6_), *δ*: 3.26 (t, *J* = 2.2 Hz, 1H, CH), 4.04 (s, 3H, NCH_3_), 4.22 (d, *J* = 2.2 Hz, 2H, SCH_2_), 8.59 (s, 1H, H-8); ^13^C NMR (DMSO-d_6_), *δ*: 18.09 (CH_2_, SCH_2_CCH), 34.84 (CH_3_, NCH_3_), 74.67 (CH, SCH_2_CCH), 79.55 (C, SCH_2_CCH), 123.00 (C, C-5), 151.09 (CH, C-8), 152.28 (C, C-2), 153.99 (C, C-6), 160.52 (C, C-4); HSQC NMR, *δ*: 8.59 (H-8) correlated with 151.09 (C-8), 4.22 (H, SCH_2_) correlated with 18.09 (C, SCH_2_), 4.04 (H, NCH_3_) correlated with 34.84 (C, NCH_3_); HMBC NMR, *δ*: 8.59 (H-8) correlated with 34.84 (C, NCH_3_), 123.00 (C-5), and 160.52 (C-4), 4.22 (H, SCH_2_) correlated with 79.55 (C, CCH), 74.67 (C, CH), and 153.99 (C-6), 4.04 (H, NCH_3_) correlated with 123.00 (C-5) and 151.09 (C-8); EIMS m/z 238 [M]^+^ (34.5), 240 [M+2] (12), 203 [M-Cl] (39), 133 [M-Cl, -SCH_2_CCH] (55), 39 [CH_2_CCH] (100); HREIMS m/z [M]^+^ calcd. for C_9_H_7_ClN_4_S 238.0079, found 238.0076.

### *Synthesis of 2*-*chloro*-*6*-*(4*-*N*-*pyrrolidinylbut*-*2*-*ynylthio)*-*7*-*methylpurine****5b***

To a mixture of propargyl derivative **5a** (0.24 g, 1 mmol) and paraformaldehyde (0.06 g, 2 mmol) in 5 ml of dry dioxane, pyrrolidine (0.14 g, 2 mmol) and CuCl (0.01 g) were added. The reaction mixture was stirred at temperature 70 °C for 2 h. After cooling, the resulting solid was filtered off and purified by column chromatography (silica gel, CHCl_3_, CHCl_3_–EtOH, 99:1 v/v) to give compound **5b**. It was obtained as a pale yellow solid (0.31 g, 96 %); mp 129–130 °C (EtOH); ^1^H NMR (CDCl_3_), *δ*: 2.13 (m, 8H, 4CH_2_), 3.12 (t, *J* = 2.2 Hz, 2H, SCH_2_), 3.62 (t, *J* = 2.2 Hz, 2H, CCH_2_), 4.19 (s, 3H, NCH_3_), 8.25 (s, 1H, H-8); ^13^C NMR (DMSO-d_6_), *δ*: 18.77 (CH_2_, SCH_2_CC), 23.68 (2CH_2_), 34.86 (CH_3_, NCH_3_), 42.58 (CH_2_, CCH_2_), 51.86 (2CH_2_, NCH_2_), 79.15 (C, SCH_2_CC), 80.01 (C, SCH_2_CC), 123.16 (C, C-5), 151.08 (CH, C-8), 152.35 (C, C-2), 154.24 (C, C-6), 160.50 (C, C-4); CIMS m/z 322 [M+1]^+^ (6.5), 324 [M+1+2] (2.5), 167 [M+1-SCH_2_CCCH_2_NC_4_H_8_] (9.5), 72 [C_4_H_8_NH_2_]^+^ (100); HRFABMS m/z [M+H]^+^ calcd. for C_14_H_17_ClN_5_S 322.0893 found 322.0893.

### *Synthesis of 2*-*chloro*-*6*-*benzylthio*-*7*-*methylpurine****6***

2-Chloro-6-benzylthio-7-methylpurine **6** was prepared from 2-chloro-7-methyl-6-purinethione **1a** (0.2 g, 1 mmol) by alkylation with benzyl chloride (0.25 g, 2 mmol) at room temperature in 4 % aqueous KOH solution (5 ml). After 30 min, the crude product was precipitated, filtered off, washed with water, and crystallized from benzene. It was obtained as a pale yellow solid (0.265 g, 91.5 %); mp 131–132 °C (benzene); ^1^H NMR (CDCl_3_), *δ*: 4.07, (s, 3H, NCH_3_), 4.64 (s, 2H, CH_2_), 7.38 (m, 5H_arom_), 7.98 (s, 1H, H-8); ^13^C NMR (DMSO-d_6_), *δ*: 33.39 (CH_2_, SCH_2_), 34.84 (CH_3_, NCH_3_), 122.72 (C, C-5), 127.98 (CH, p-CH), 128.99 (2CH, m-CH), 129.84 (2CH, o-CH), 137.12 (C, CCH_2_), 150.79 (CH, C-8), 152.26 (C, C-2), 155.46 (C, C-6), 160.33 (C, C-4); EIMS m/z 290 [M]^+^ (100), 292 [M+2] (28.5), 199 [M-C_6_H_5_CH_2_] ^+^ (87); HREIMS m/z [M^+^] calcd. for C_13_H_11_ClN_4_S 290.0392 found 290.0385.

### Chlorination and amination of 2-chloro-6-benzylthio-7-methylpurine 6

The chlorinolysis was carried out by passing chlorine gas into a stirred mixture composed of 2-chloro-6-benzylthio-7-methylpurine **6** (0.29 g, 1 mmol), 3 ml of chloroform, and 3 ml of 80 % acetic acid cooled at 5 °C. The passage of chlorine gas was continued for 30 min, and then the mixture was poured into 10 ml of ice water. The chloroform layer was separated, and aqueous layer was extracted with chloroform (2 × 5 ml). The chloroform extracts were combined, washed with water, dried over anhydrous sodium sulfate, and evaporated *in vacuo* to give an oil residue. In order to remove benzyl acetate and benzyl chloride, the residue was triturated with ice-cold dry ether and the ether layer was separated. The mixture of obtained oil (0.26 g, 1 mmol), and 25 % aqueous ammonia (2.5 ml) or diethylamine (0.22 g, 3 mmol) in 10 % NaOH solution or aniline (0.19 g, 2 mmol) in 2.5 ml of benzene was stirred at 40–45 °C for 2–6 h. The resulted solid was filtered off (in the case of the reaction with ammonia, the excess of ammonia was removed in vacuo and the residue was diluted with water up to volume 2.5 ml) to give compounds **9a–c**. Products were purified by a column chromatography (aluminum oxide, CHCl_3_–EtOH, 99:1 v/v) to give:

#### *2-Chloro-6-amine-7-methylpurine****9a***

was obtained as a white solid (0.15 g, 82 %); mp 248–249 °C (EtOH), lit. mp 250 °C (Adams and Whitmore, 1945); ^1^H NMR (DMSO-d_6_), *δ*: 3.97 (s, 3H, NCH_3_), 7.46 (s, 2H, NH_2_), 8.20 (s, 1H, H-8); ^13^C NMR (DMSO-d_6_), *δ*: 34.29 (CH_3_, NCH_3_), 111.12 (C, C-5), 147.27 (CH, C-8), 153.03 (C, C-2), 153.17 (C, C-6), 161.50 (C, C-4); EIMS m/z 183 [M]^+^ (100), 185 [M+2] (29); HREIMS m/z [M]^+^ calcd. for C_6_H_6_ClN_5_ 183.0311 found 183.0307.

#### *2-Chloro-6-diethylamine-7-methylpurine****9b***

was obtained as a white solid (0.20 g, 84 %); mp 106–107 °C (EtOH), lit. mp 107 °C (Adams and Whitmore, 1945); ^1^H NMR (DMSO-d_6_), *δ*: 1.17 (t, *J* = 7.7 Hz, 6H, 2 × CH_3_CH_2_N), 3.52 (q, *J* = 7.7 Hz, 4H, 2 × CH_3_ CH_2_N), 3 0.94 (s, 3H, NCH_3_), 8.37 (s, 1H, H-8); ^13^C NMR (DMSO-d_6_), *δ*: 12.93 (2CH_3_, NCH_2_CH_3_), 35.63 (CH_3_, NCH_3_), 44.32 (2CH_2_, NCH_2_CH_3_), 114.45 (C, C-5), 149.30 (CH, C-8), 151.81 (C, C-2), 154.70 (C, C-6), 163.03 (C, C-4); EIMS m/z 239 [M]^+^ (19), 241 [M+2] (6); 210 [M-C_2_H_5_], (100); HREIMS m/z [M]^+^ calcd. for C_10_H_14_ClN_5_ 239.0937 found 239.0935.

#### *2-Chloro-6-phenylamine-7-methylpurine****9c***

was obtained as a white solid (0.18 g, 69 %); mp 202–203 °C. (EtOH); ^1^H NMR (CDCl_3_), *δ*: 3.96 (s, 3H, NCH_3_), 6.84 (s, 1H, NH), 7.23 (t, *J* = 7.20 Hz, 1H, p-C_6_H_5_), 7.40 (t, *J* = 7.20 Hz, 2H, m-C_6_H_5_), 7.52 (d, *J* = 7.20 Hz, 2H, o-C_6_H_5_), 7.90 (s, 1H, H-8); ^13^C NMR (DMSO-d_6_), *δ*: 34.63 (CH_3_, NCH_3_), 112.40 (C, C-5), 123.17 (2CH, m-CH), 124.61 (CH, p-CH), 129.06 (2CH, o-CH), 138.97 (C, CNH), 148.47 (CH, C-8), 149.75 (C, C-2), 152.29 (C, C-6), 162.07 (C, C-4); FABMS m/z 260 [M+1]^+^ (100), 262 [M+1+2] (31.5); HREIMS m/z [M]^+^ calcd. for C_12_H_10_ClN_5_ 259.0625 found 259.0626.

### General synthesis of 2-substituted 7-methyl-6-purinesulfenamide 10a–e

A solution of commercial NaOCl (14.5 %, 1.7 ml) and 1.7 ml of water was cooled to 0 °C in an ice–NaCl bath. Ammonium hydroxide (25 %, 2 ml) was similarly cooled and added with stirring to the bleach solution. The mixture was stirred at −5  to 0 °C for 15 min and then allowed to cool to 0 °C solution of 2-substituted 7-methyl-6-purinethione **1a–d** (2 mmol) in 10 % KOH (5 ml) was dropped. The mixture was stirred in stoppered flask for 30 min at temperature −5  to 0 °C. The reaction mixture was initially clear, light yellow solution, but after 30 min a solid began separating. After allowing the reaction mixture to stand at room temperature for an additional 1 h, the precipitated product was filtered off and washed with a small amount of cold water, followed by cold ethanol to yield purine 6-sulfenamide **10a–c, 10e**. The crude products were purified by a column chromatography (silica gel, CHCl_3_, CHCl_3_–EtOH, 99:1 v/v). The aqueous filtrate separated after precipitation of product **10e** was evaporated to dryness and extracted with absolute ethanol (3 × 5 ml). The ethanolic solvent was evaporated, and the residue was purified by column chromatography (aluminum oxide, CHCl_3_–EtOH, 10:1 v/v) to give compound **10d**.

#### *2-Chloro-7-methyl-6-purinesulfenamide****10a***

was obtained as a yellow solid (0.35 g, 87.5 %); mp 149–150 °C (EtOH); ^1^H NMR (DMSO-d_6_), *δ*: 3.98 (s, 3H, NCH_3_), 4.41 (s, 2H, NH_2_), 8.51 (s, 1H, H-8); ^13^C NMR (DMSO-d_6_), *δ*: 35.13 (CH_3_, NCH_3_), 120.85 (C, C-5), 150.41 (CH, C-8), 152.99 (C, C-4), 159.88, 164.43 (C, C-2, and C-6); FABMS m/z 216 [M+1]^+^ (38), 218 [M+1+2] (12), 185 (100); HREIMS m/z [M]^+^ calcd. for C_6_H_6_ClN_5_S 215.0032 found 215.0029.

#### *2-Methoxy-7-methyl-6-purinesulfenamide****10b***

was obtained as a yellow solid (0.39 g, 92.5 %); mp 158–159 °C (EtOH); ^1^H NMR (DMSO-d_6_), *δ*: 3.93 (s, 6H, OCH_3_, NCH_3_), 4.20 (s, 2H, NH_2_), 8.30 (s, 1H, H-8); ^13^C NMR (DMSO-d_6_), *δ*: 34.80 (CH_3_, NCH_3_), 54.81 (CH_3_, OCH_3_), 117.79 (C, C-5), 148.81 (CH, C-8), 160.55 (C, C-4), 161.72, 162.98 (C, C-2, and C-6); FABMS m/z 212 [M+1]^+^ (100); HREIMS m/z [M]^+^ calcd. for C_7_H_9_N_5_OS 211.0527 found 211.0523.

#### *2-Methylthio-7-methyl-6-purinesulfenamide****10c***

was obtained as a yellow solid (0.385 g, 85 %); mp 164–165 °C (EtOH); ^1^H NMR (DMSO-d_6_), *δ*: 2.57 (s, 3H, SCH_3_), 3.94 (s, 3H, NCH_3_), 4.25 (s, 2H, NH_2_), 8.35 (s, 1H, H-8); ^13^C NMR (DMSO-d_6_), *δ*: 14.32 (CH_3_, SCH_3_), 34.86 (CH_3_, NCH_3_), 119.06 (C, C-5), 151.57 (CH, C-8), 159.32 (C, C-4), 161.52, 164.01 (C, C-2, and C-6); FABMS m/z 228 [M+1]^+^ (100); HREIMS m/z [M]^+^ calcd. for C_7_H_9_N_5_S_2_ 227.0299 found 227.0299.

#### *7-Methyl-6-sulfenamide-2-purinethione****10d***

was obtained as a yellow solid (0.07 g, 16.5 %); mp 254–256 °C (EtOH); ^1^H NMR (DMSO-d_6_), *δ*: 4.20(s, 3H, NCH_3_), 4.37 (s, 2H, NH_2_), 8.18 (s, 1H, H-8), 10.83 (s, 1H, NH); ^13^C NMR (DMSO-d_6_), *δ*: 34.68 (CH_3_, NCH_3_), 121.30 (C, C-5), 147.96 (CH, C-8), 157.40 (C, C-4), 169.22, 173.95 (C, C-2, and C-6); FABMS m/z 214 [M+1] (100); HREIMS m/z [M]^+^ calcd. for C_6_H_7_N_5_S_2_ 213.0143 found 213.0141.

#### *7-Methyl-2,6-purinedisulfenamide****10e***

was obtained as a yellow solid (0.315 g, 69 %); mp 185–186 °C (EtOH); ^1^H NMR (DMSO-d_6_), *δ*: 3.94(s, 3H, NCH_3_), 4.03 (s, 2H, NH_2_), 4.18 (s, 2H, NH_2_), 8.35 (s, 1H, H-8); ^13^C NMR (DMSO-d_6_), *δ*: 34.82(CH_3_, NCH_3_), 119.09 (C, C-5), 148.72 (CH, C-8), 159.45 (C, C-4), 161.26, 169.60 (C-2 and C-6); HSQC NMR, *δ*: 8.35 (H-8) correlated with 148.72 (C-8), 3.94 (H, NCH_3_) correlated with 34.82 (C, NCH_3_); HMBC NMR, *δ*: 8.35 (H-8) correlated with 34.82 (C, NCH_3_), 119.09 (C-5), and 159.45 (C-4), 3.94 (H, NCH_3_) correlated with 119.09 (C-5) and with 148.72 (C-8); FABMS m/z 229 [M+1] (100); HREIMS m/z [M]^+^ calcd. for C_6_H_8_N_6_S_2_ 228.0252 found 228.0255.

### General synthesis of 2-substituted 7-methyl-6-purinesulfonamide 8a–c, 8e

A solution of 3-chloroperbenzoic acid (MCPBA) (77 %, 0.86 g, 4 mmol) in 10 ml of ethanol was added dropwise for 10 min to a solution of sulfonamides **10a–c** and **10e** (1 mmol) in 79 ml of absolute ethanol. The mixture was stirred at room temperature for 1.5 h, and a precipitated solid was filtered off to give crude products **8a–c** and **8e**. The ethanolic filtrate was evaporated to dryness and extracted with ether to remove 3-chlorobenzoic acid. The solid residue together with crude product was purified by a column chromatography (aluminum oxide, CHCl_3_, CHCl_3_–EtOH, 9:1 v/v) to give also compounds **8a–c**, **8e**.

#### *2-Chloro-7-methyl-6-purinesulfonamide****8a***

was obtained as a white solid (0.18 g, 73 %); mp 174–175 °C (EtOH); ^1^H NMR (DMSO-d_6_), *δ*: 4.07 (s, 3H, NCH_3_), 6.91 (s, 2H, NH_2_), 8.82 (s, 1H, H-8); ^13^C NMR (DMSO-d_6_), *δ*: 36.46 (CH_3_, NCH_3_), 121.55 (C, C-5), 151.93 (CH, C-8), 153.88 (C, C-4), 160.39, 164.62 (C, C-2, and C-6); EIMS m/z 247 [M]^+^ (1.5), 249 [M+2] (0.5); 183 [M-SO_2_] (14), 168 [M-SO_2_NH_2_] (100); HREIMS m/z [M]^+^ calcd. for C_6_H_6_ClN_5_O_2_S 246.9930 found 246.9927.

#### *2-Methoxy-7-methyl-6-purinesulfonamide****8b***

was obtained as a white solid (0.185 g, 76 %); mp 203–204 °C. (EtOH); ^1^H NMR (DMSO-d_6_), *δ*: 3.88 (s, 3H, OCH_3_), 4.04 (s, 3H, NCH_3_), 7.99 (s, 2H, NH_2_), 8.70 (s, 1H, H-8); ^13^C NMR (DMSO-d_6_), *δ*: 35.86 (CH_3_, NCH_3_), 55.59 (CH_3_, OCH_3_), 117.32 (C, C-5), 133.80 (CH, C-8), 153.60 (C, C-4), 157.55, 166.54 (C, C-2, and C-6); EIMS m/z 243 [M]^+^ (26), 180 [M-SO_2_] (55.5), 162 [M-SO_2_NH_2_] (8), 148 (100); HREIMS m/z [M]^+^ calcd. for C_7_H_9_N_5_O_3_S 243.0426 found 243.0419.

#### *2-Methylthio-7-methyl-6-purinesulfonamide****8c***

was obtained as a white solid (0.17 g, 66 %); mp 198–199 °C (EtOH); ^1^H NMR (DMSO-d_6_), *δ*: 2.56 (s, 3H, SCH_3_), 4.03 (s, 3H, NCH_3_), 7.86 (s, 2H, NH_2_), 8.75 (s, 1H, H-8); ^13^C NMR (DMSO-d_6_), *δ*: 15.59 (CH_3_, SCH_3_), 34.04 (CH_3_, NCH_3_), 116.45 (C, C-5), 129.28 (CH, C-8), 133.20 (C, C-4), 147.01, 166.54 (C, C-2, and C-6); CIMS m/z 260 [M+1] (22), 196 [M+1-SO_2_] (64), 180 (M+1-SO_2_NH_2_] (100); HREIMS m/z [M]^+^ calcd. for C_7_H_9_N_5_O_2_S_2_ 259.0198 found 259.0195.

#### *7-Methyl-2,6-purinedisulfonamide****8d***

was obtained as a white solid (0.23 g, 79 %); mp 214–215 °C (EtOH); ^1^H NMR (DMSO-d_6_), *δ*: 4.17(s, 3H, NCH_3_), 7.01 (s, 2H, NH_2_), 7.69 (s, 2H, NH_2_), 8.97 (s, 1H, H-8); ^13^C NMR (DMSO-d_6_), *δ*: 36.30 (CH_3_, NCH_3_), 122.52 (C, C-5), 154.76 (CH, C-8), 158.11 (C, C-4), 159.98, 162.99 (C, C-2, and C-6); EIMS m/z 292 [M]^+^ (9), 229 (M-SO_2_] (7), 149 [M-SO_2_ NH_2_] (22), 57 (100); HREIMS m/z [M]^+^ calcd. for C_6_H_8_N_6_O_4_S_2_ 292.0048 found 292.0044.

### Antiproliferative assay in vitro

#### Cell culture

Compounds were evaluated for their anticancer activity using three cultured cell lines: SNB-19 (human glioblastoma, DSMZ—German Collection of Microorganisms and Cell Cultures, Braunschweig, Germany), C-32 (human amelanotic melanoma, ATCC—American Type Culture Collection, Manassas, VA, USA), T47D (human ductal breast epithelial tumor cell line, ATCC, Manassas, VA, USA), and HFF-1 (human fibroblast cell line, ATCC, Manassas, VA, USA). The cultured cells were kept at 37 °C and 5 % CO_2_. The cells were seeded (1 × 10^4^ cells/well/100 μl DMEM supplemented with 10 % FCS and streptomycin and penicillin) using 96-well plates (Corning).

#### Proliferation

In recent years, tetrazolium salts have been described which can be used for the measurement of cell proliferation and viability. The tetrazolium salts are cleaved to formazan by cellular enzymes. An expansion in the number of viable cells results in an increase in the overall activity of mitochondrial dehydrogenases in the sample. This augmentation in enzyme activity leads to an increase in the amount of formazan dye formed, which directly correlates with the number of metabolically active cells in the culture. The formazan dye produced by metabolically active cells is quantified by a scanning ELISA reader by measuring the absorbance of the dye solution at appropriate wavelengths (*λ* = 420–480 nm with a reference wavelength *λ* = 600 nm).

#### WST-1 assay

Antiproliferative effect of compounds was determined using the Cell Proliferation Reagent WST-1 assay (Roche Diagnostics, Mannheim, Germany). After exposure to tested compounds (at concentrations between 0 and 100 μg/ml) for 72 h, cells were incubated with WST-1 (10 μl) for 1 h, and the absorbance of the samples against a background control was read at 450 nm using with a reference wavelength *λ* = 600 nm a microplate reader. Results are expressed as means of at least two independent experiments performed in triplicate.

## Electronic supplementary material

Below is the link to the electronic supplementary material.

Supplementary material 1 (PDF 5021 kb)
