# Supplementary material for: Synthesis and anticancer activity of thiosubstituted purines
Source: Med Chem Res. 2015 Mar 25;24(7):3107–16. doi: 10.1007/s00044-015-1364-2 (PMC4491105; doi:10.1007/s00044-015-1364-2)

SYNTHESIS AND ANTICANCER ACTIVITY OF THIOSUSTITUTED PURINES

Alicja Kowalska[1]*, Małgorzata Latocha[2] and Krystian Pluta[1]

[1]The Medical University of Silesia, School of Pharmacy with the Division of Laboratory Medicine, Department of Organic Chemistry, Jagiellońska 4, 41-200 Sosnowiec, Poland,

[2]The Medical University of Silesia, School of Pharmacy with the Division of Laboratory Medicine, Department of Cell Biology, Jedności 8, 41-200 Sosnowiec, Poland

NMR and MS spectra

A. ^{1}H NMR, ^{13}C NMR

1. Compound **5a**
2. Compound **5b**
3. Compound **6**
4. Compound **8a**
5. Compound **8b**
6. Compound **8d**
7. Compound **9a**
8. Compound **9b**
9. Compound **9c**
10. Compound **10a**
11. Compound **10b**
12. Compound **10c**
13. Compound **10d**
14. Compound **10e**

B. 2D NMR: HSQC and HMBC

1. Compound **5a**
2. Compound **10e**

Compound **5a** ^{1}H NMR

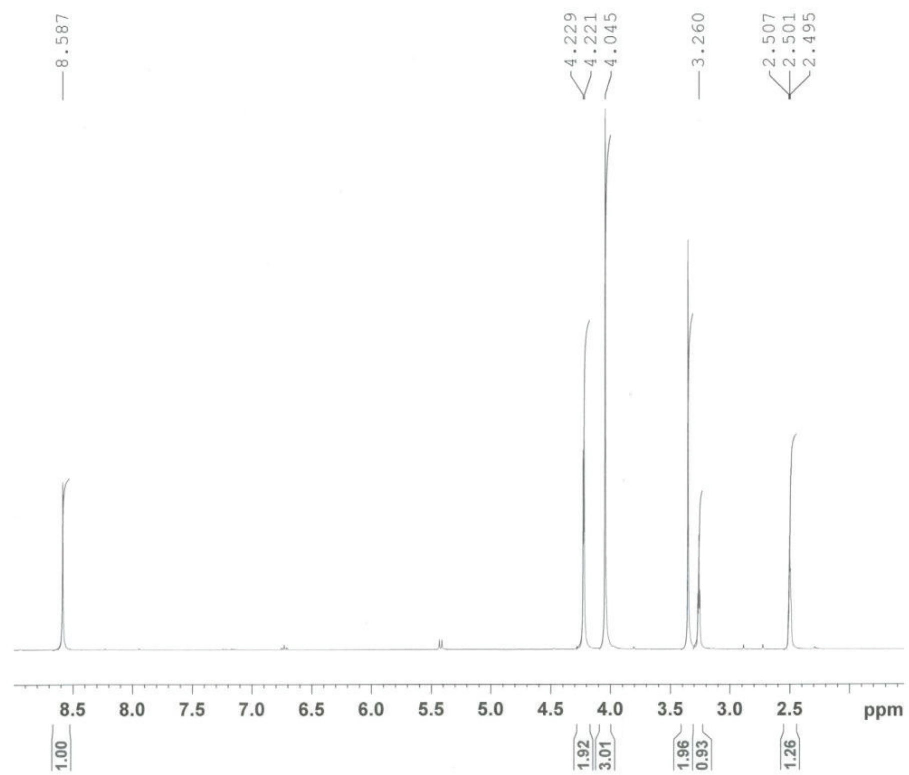

Compound **5a** ^{13}C NMR

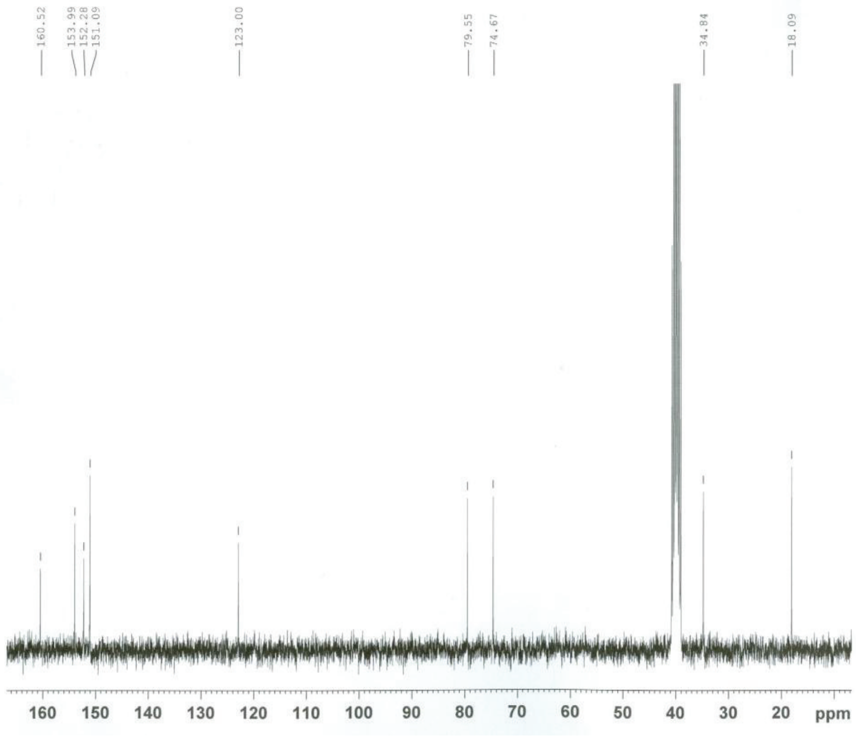

Compound **5b** ^{1}H NMR

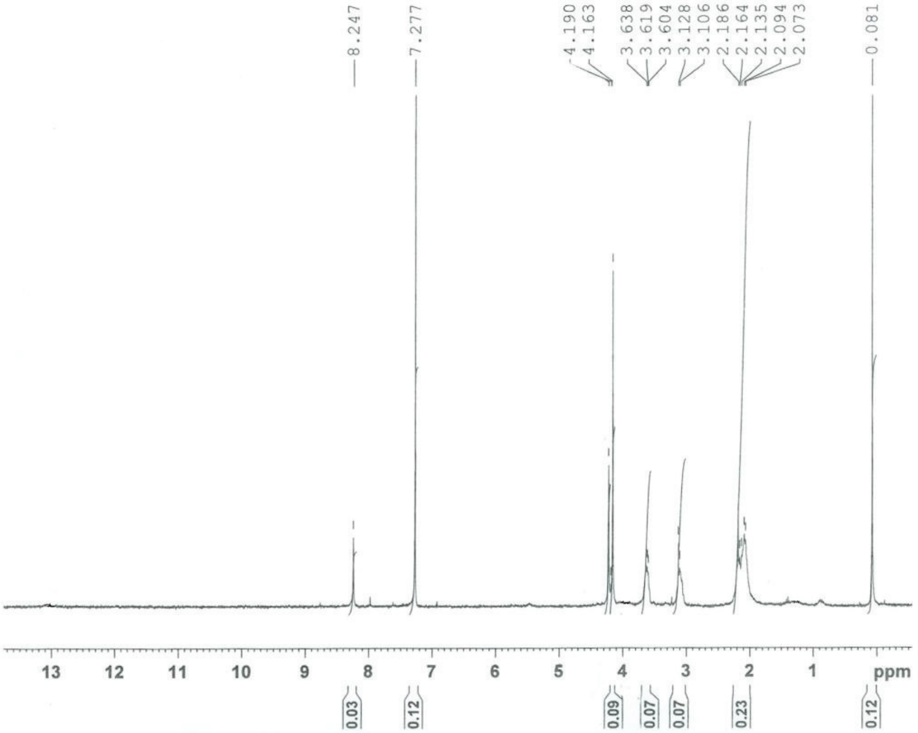

Compound **5b** ^{13}C NMR

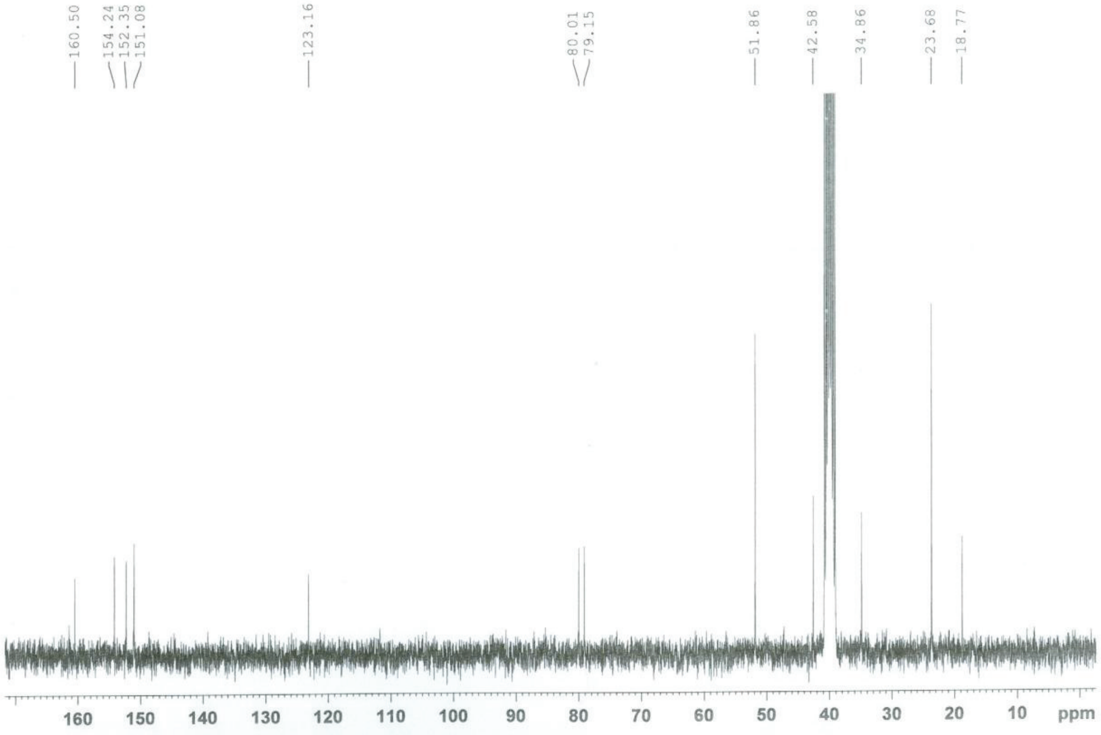

Compound **6** ^{1}H NMR

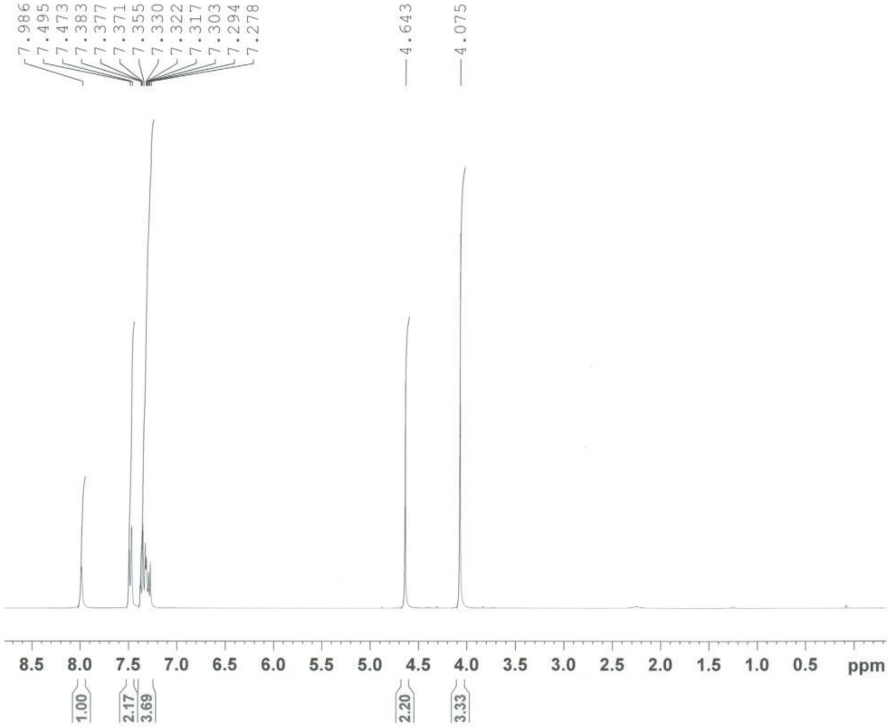

Compound **6** ^{13}C NMR

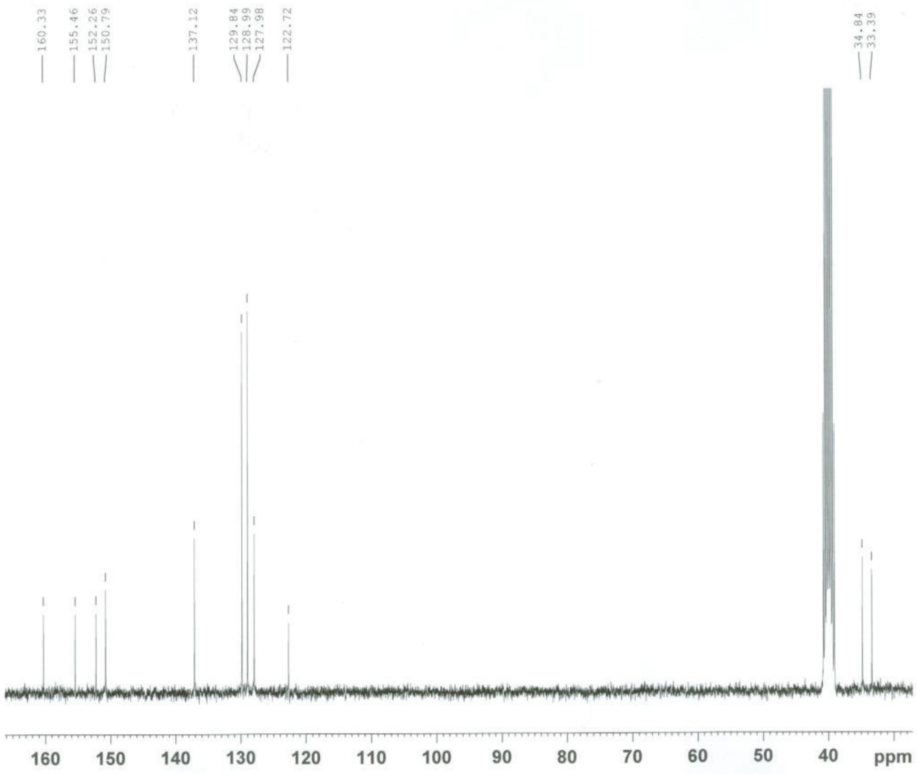

Compound **8a** ^{1}H NMR

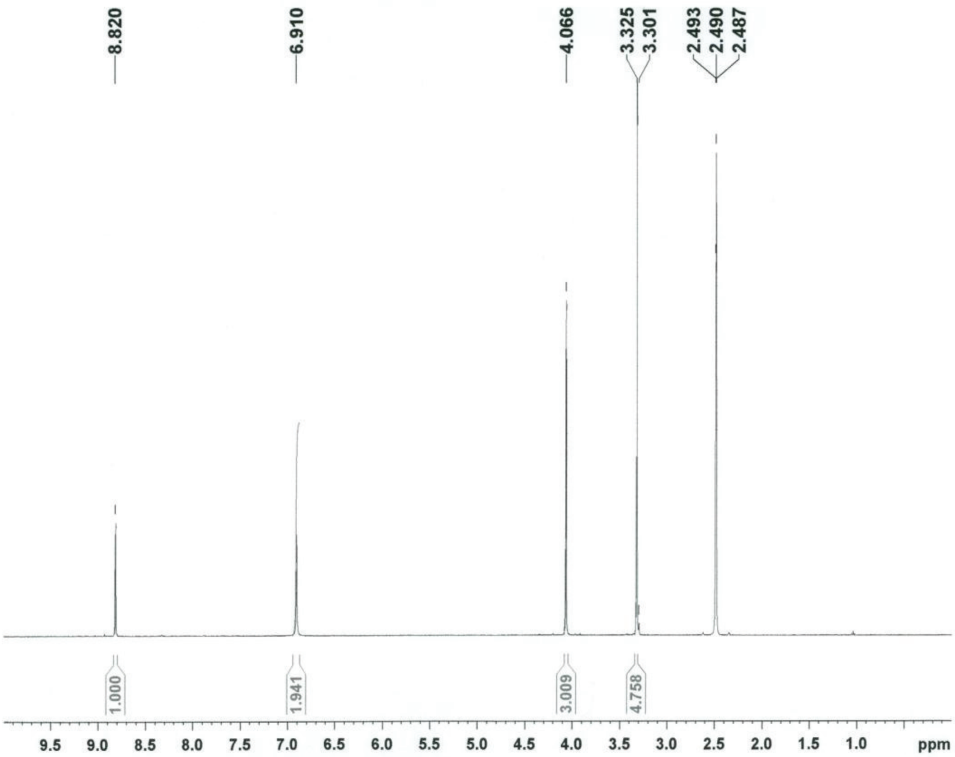

Compound **8a** ^{13}C NMR

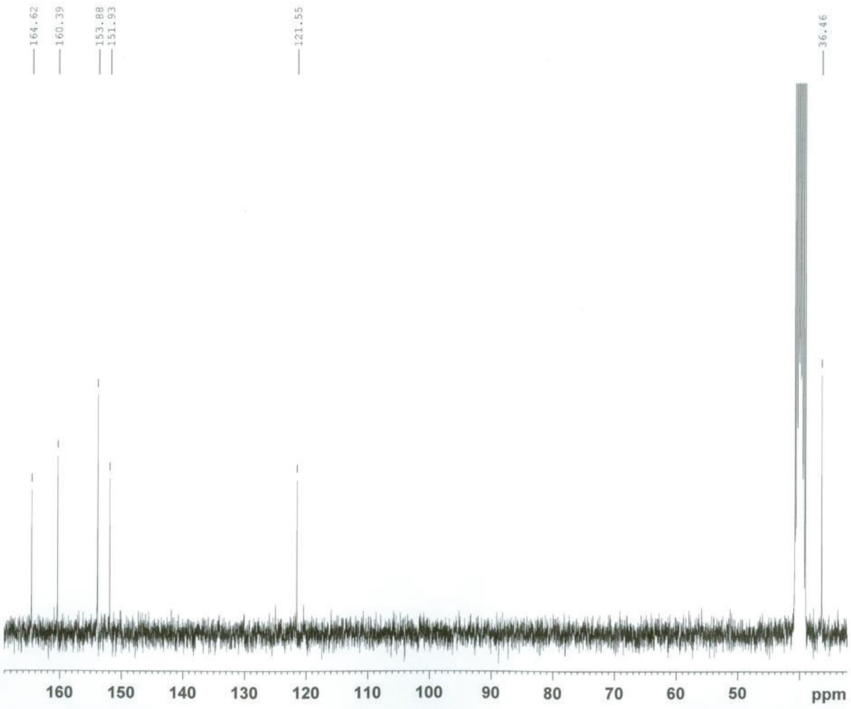

Compound **8b** ^{1}H NMR

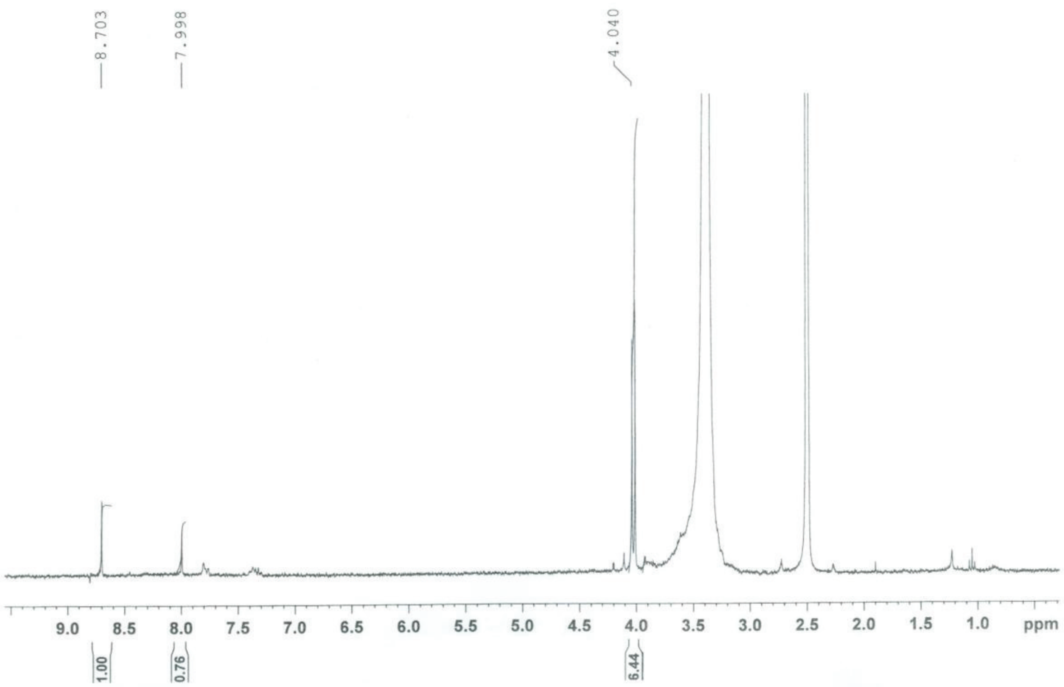

Compound **8b** ^{13}C NMR

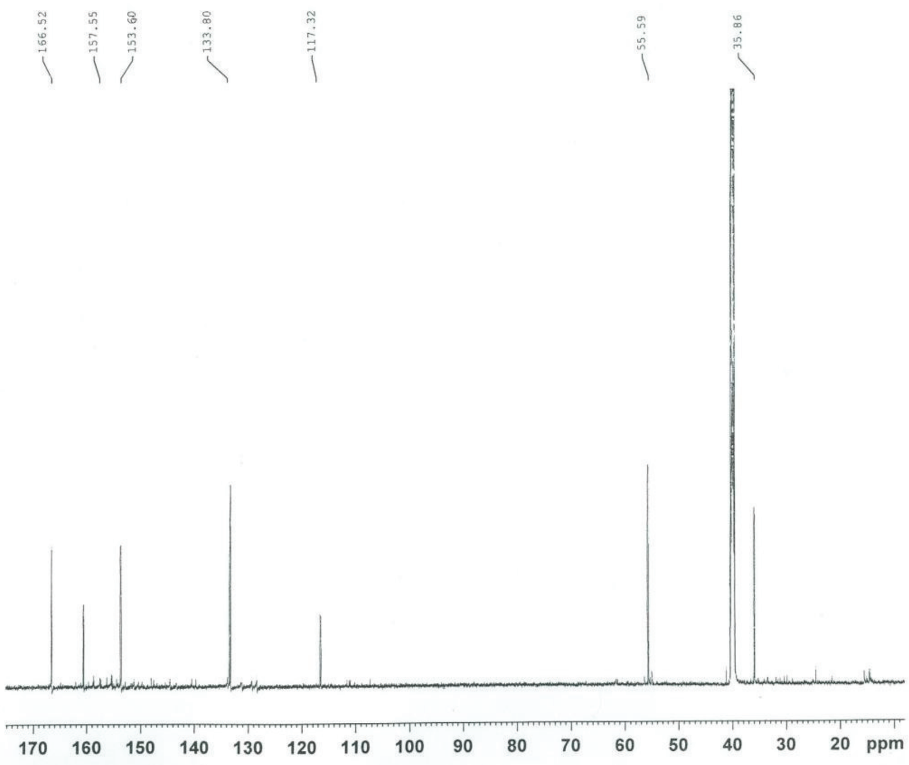

Compound **8d** ^{1}H NMR

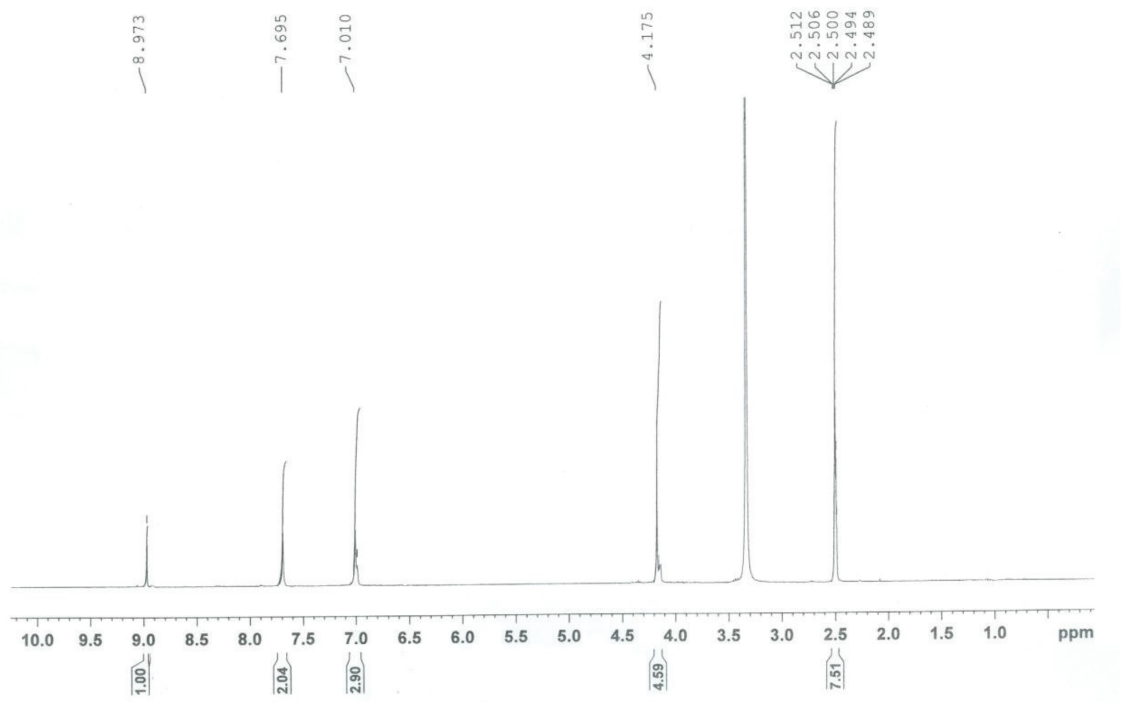

Compound **8d** ^{13}C NMR

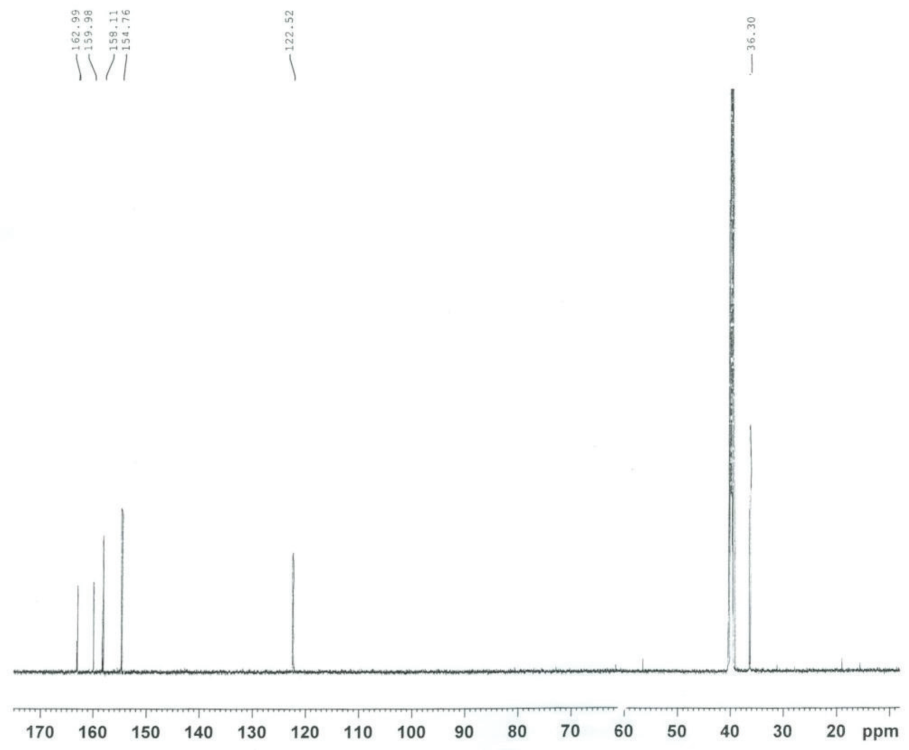

Compound **9a** ^{1}H NMR

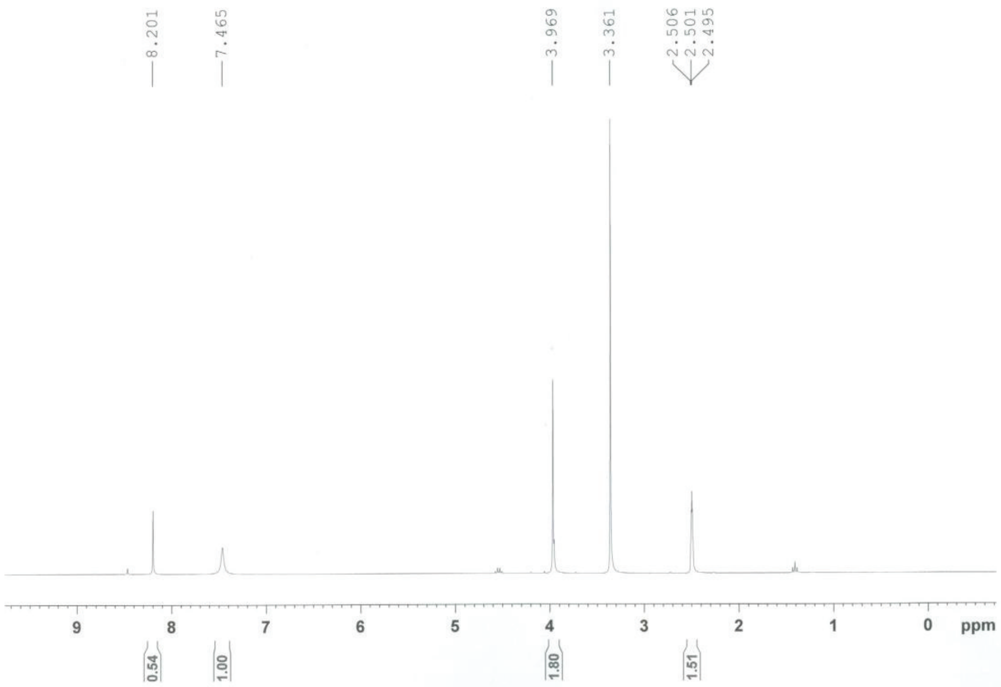

Compound **9a** ^{13}C NMR

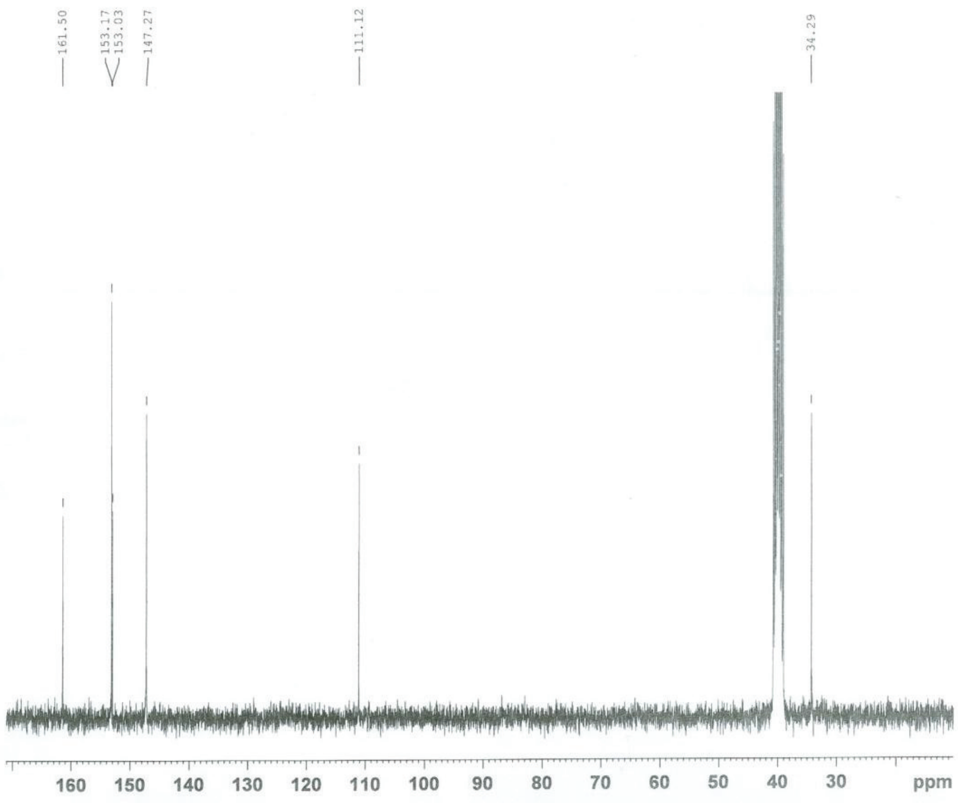

Compound **9b** ^{1}H NMR

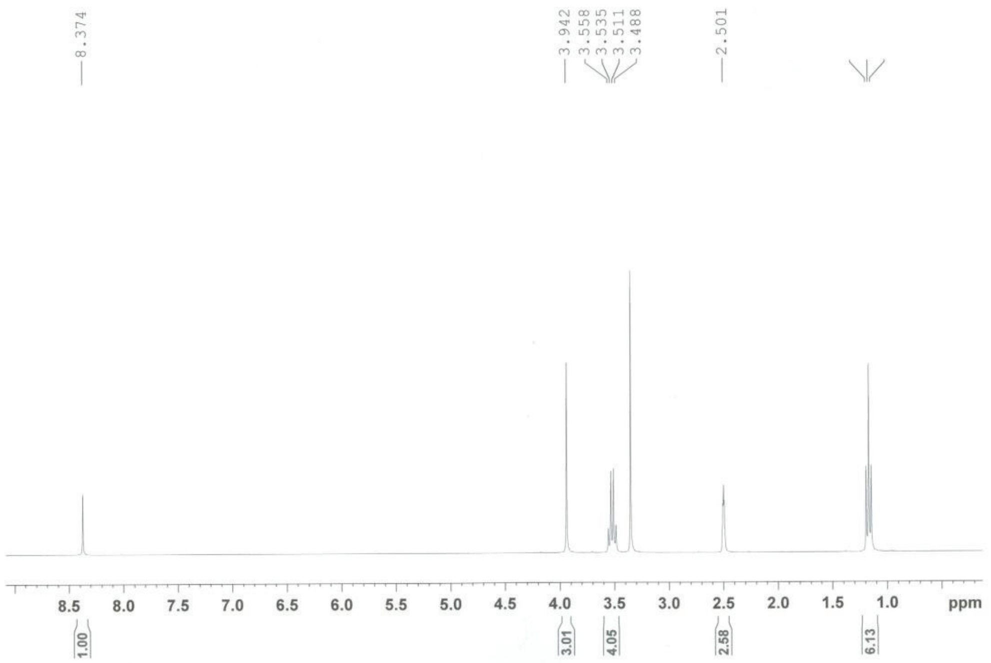

Compound **9b** ^{13}C NMR

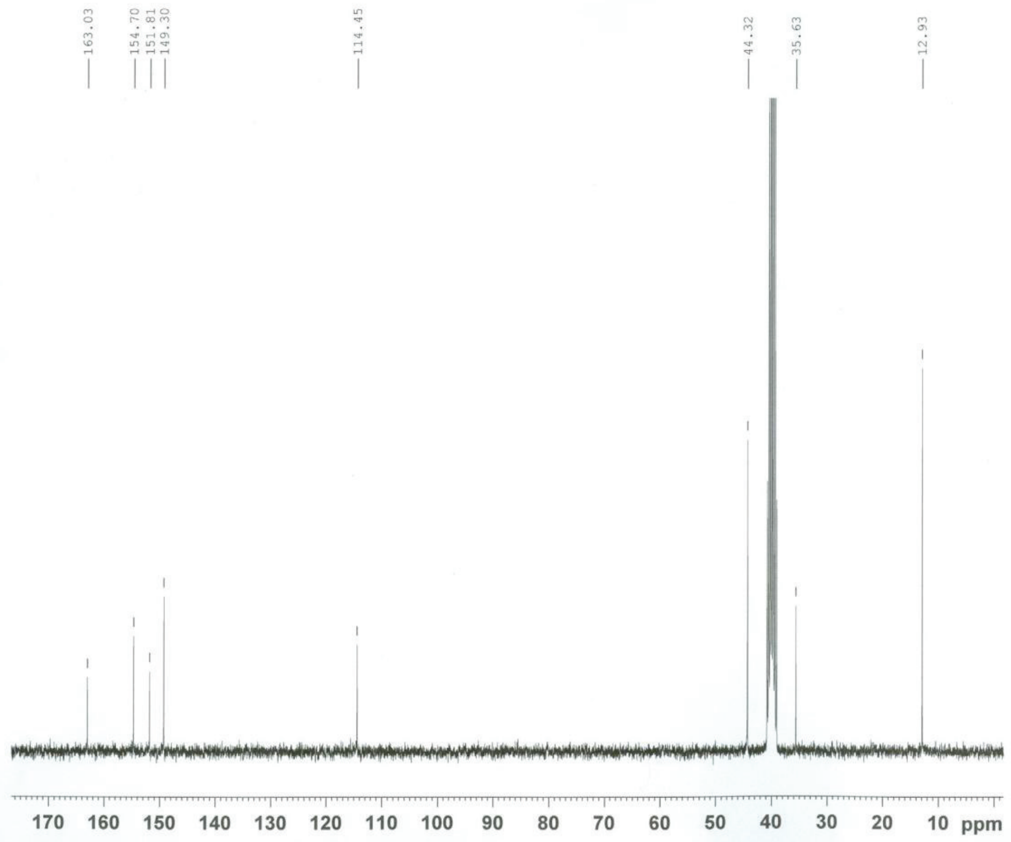

Compound **9c** ^{1}H NMR

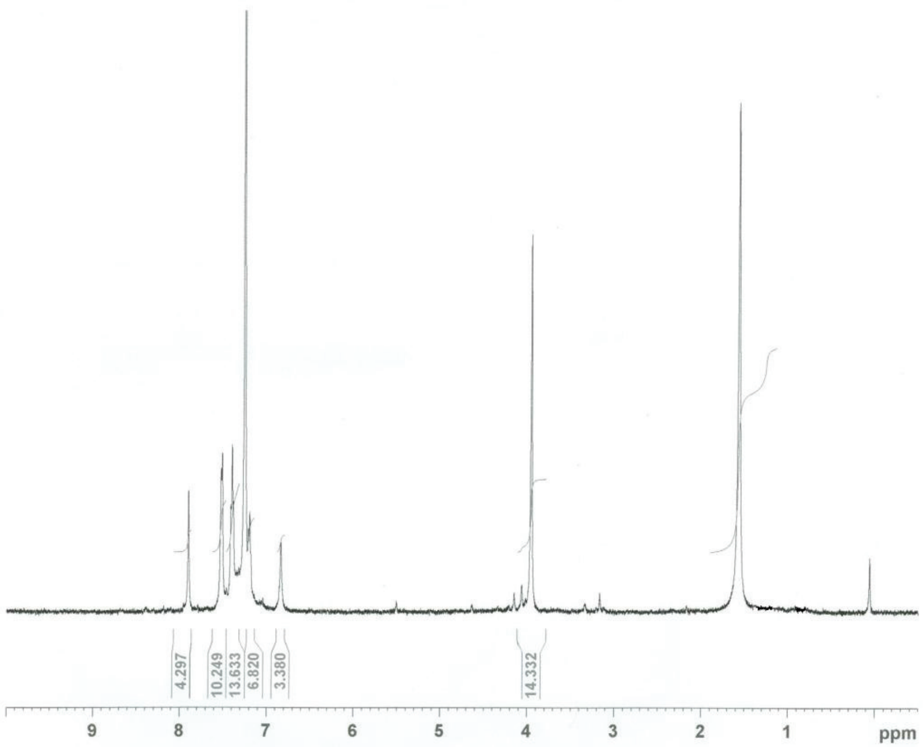

Compound **9c** ^{13}C NMR

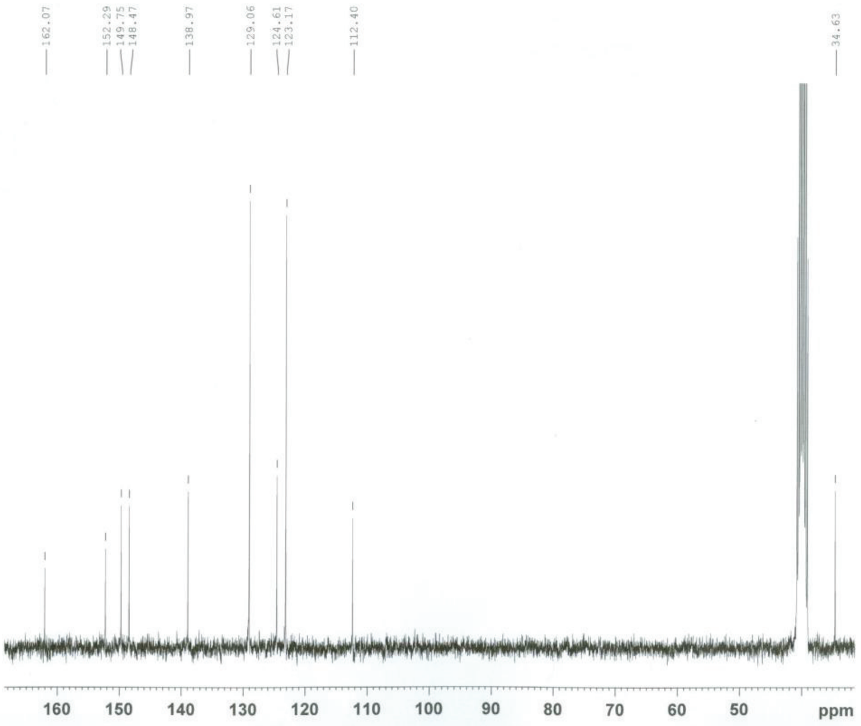

Compound **10a** ^{1}H NMR

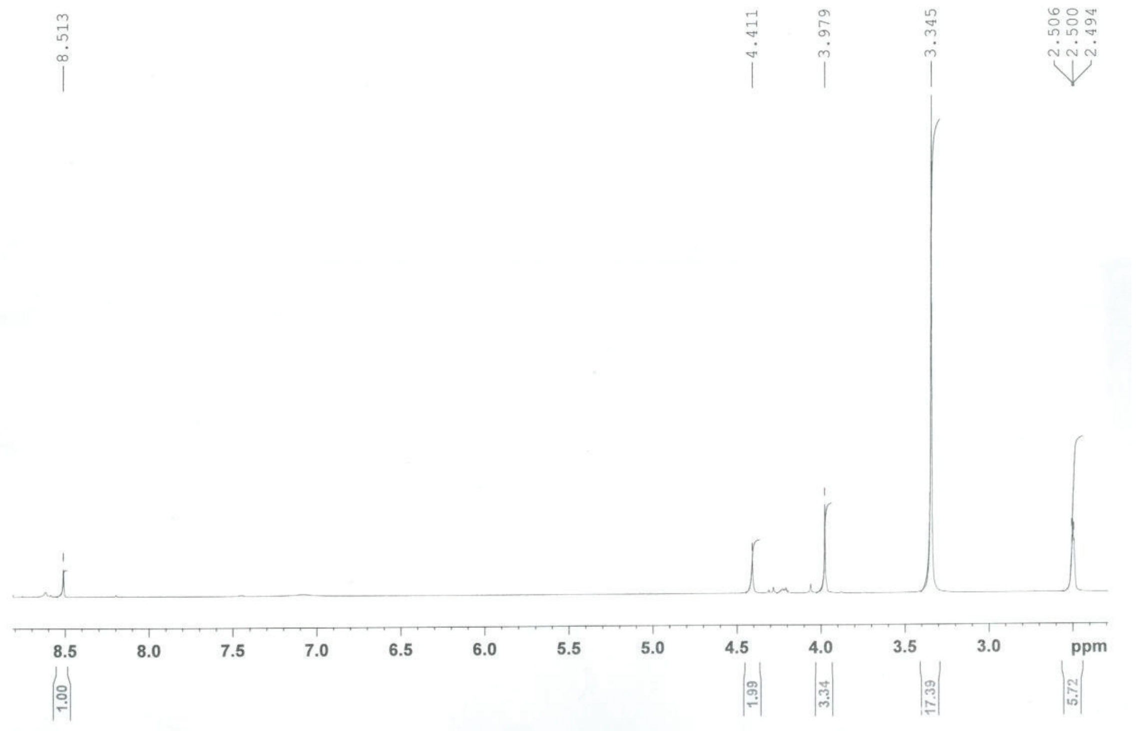

Compound **10a** ^{13}C NMR

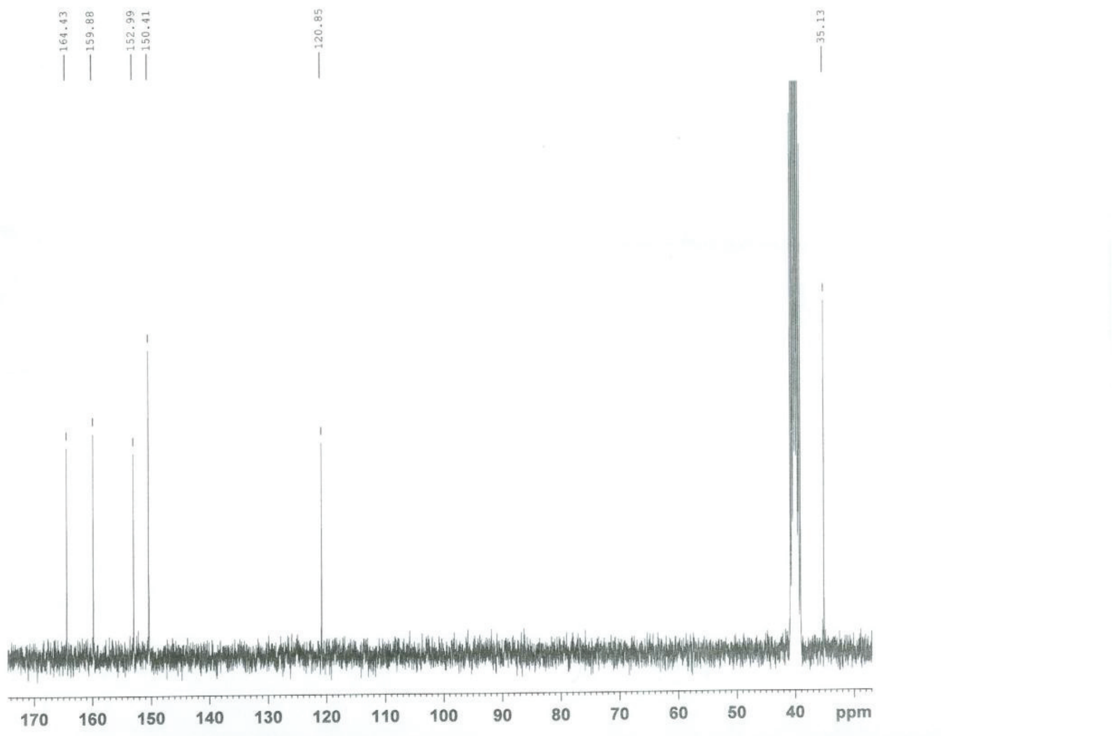

Compound **10b** ^{1}H NMR

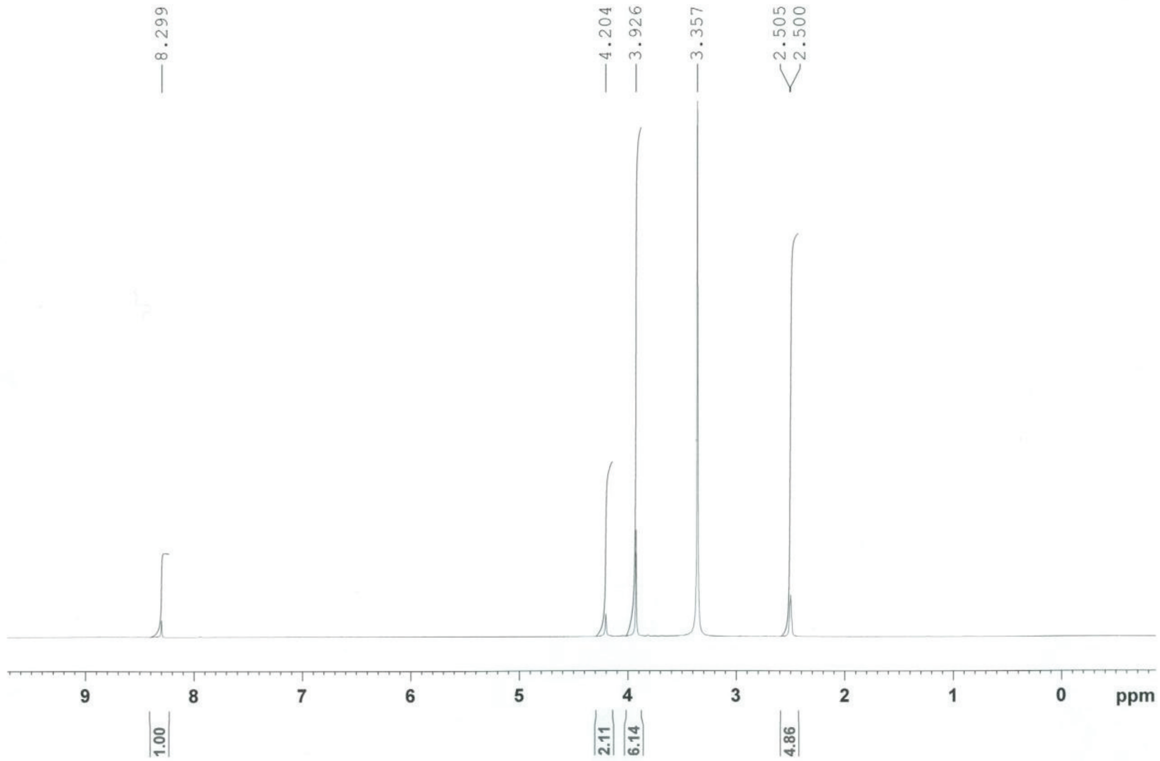

Compound **10b** ^{13}C NMR

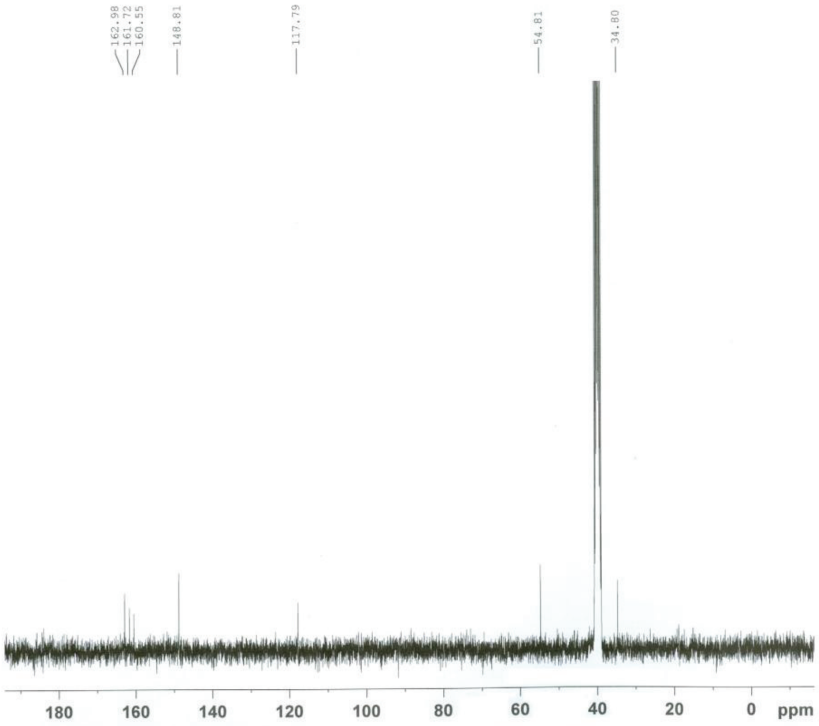

Compound **10c** ^{1}H NMR

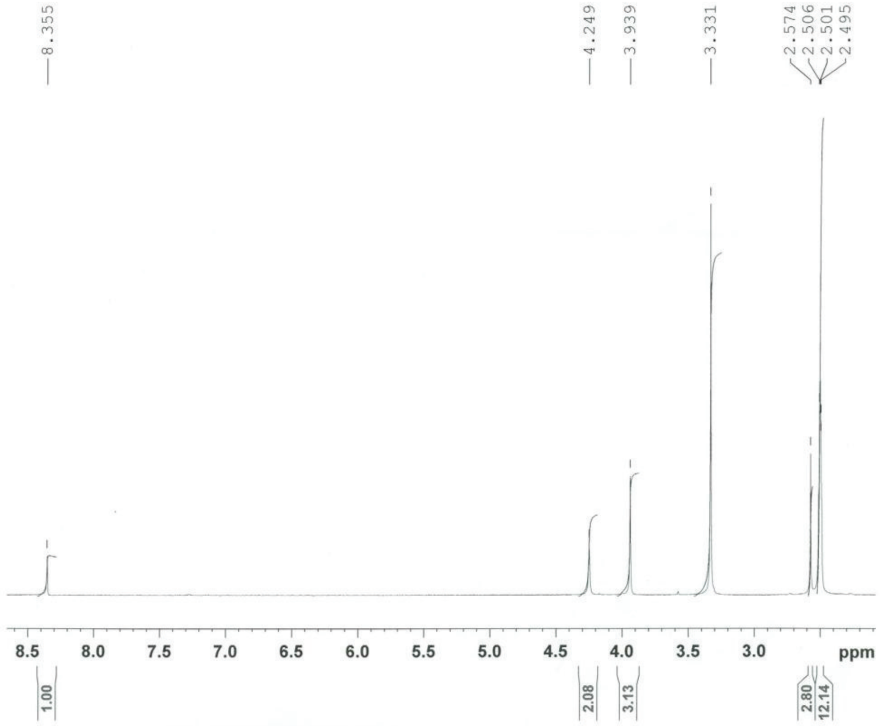

Compound **10c** ^{13}C NMR

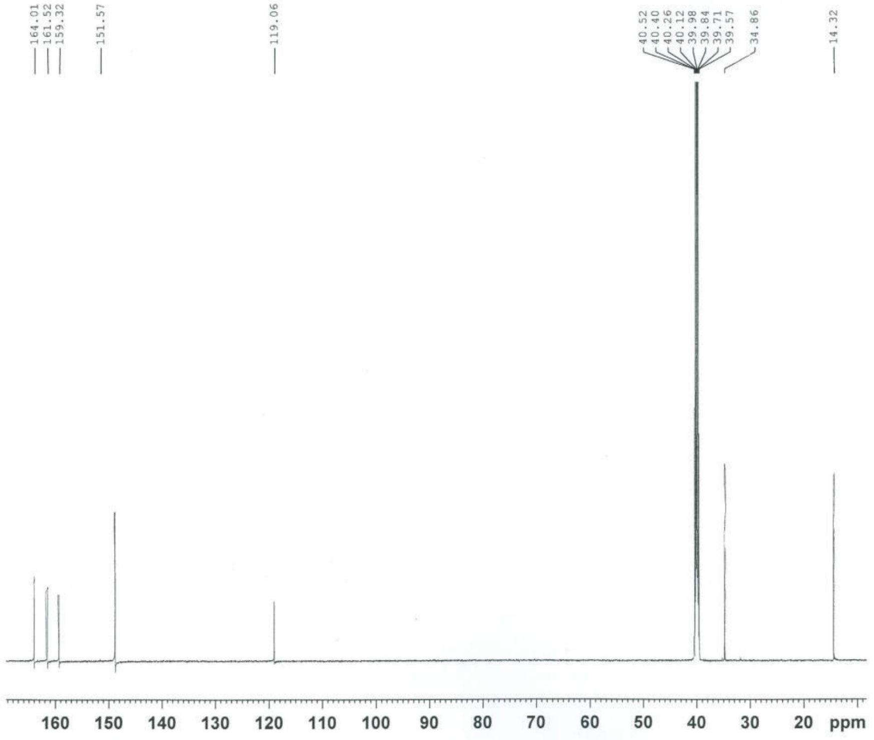

Compound **10d** ^{1}H NMR

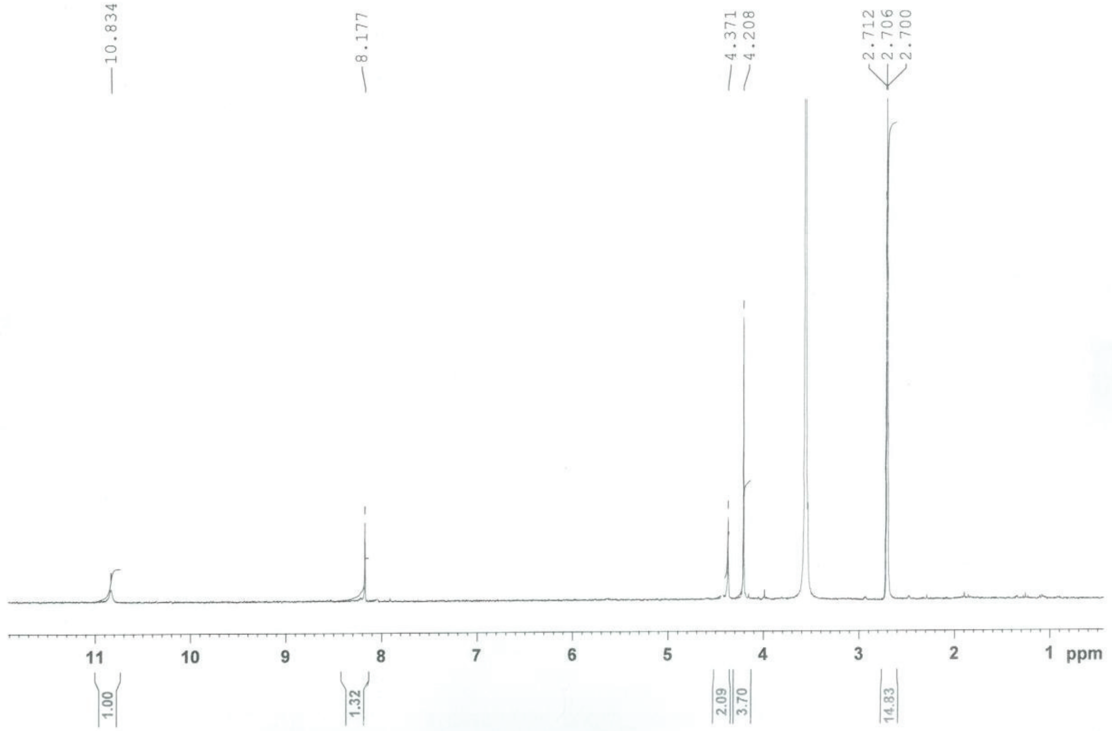

Compound **10d** ^{13}C NMR

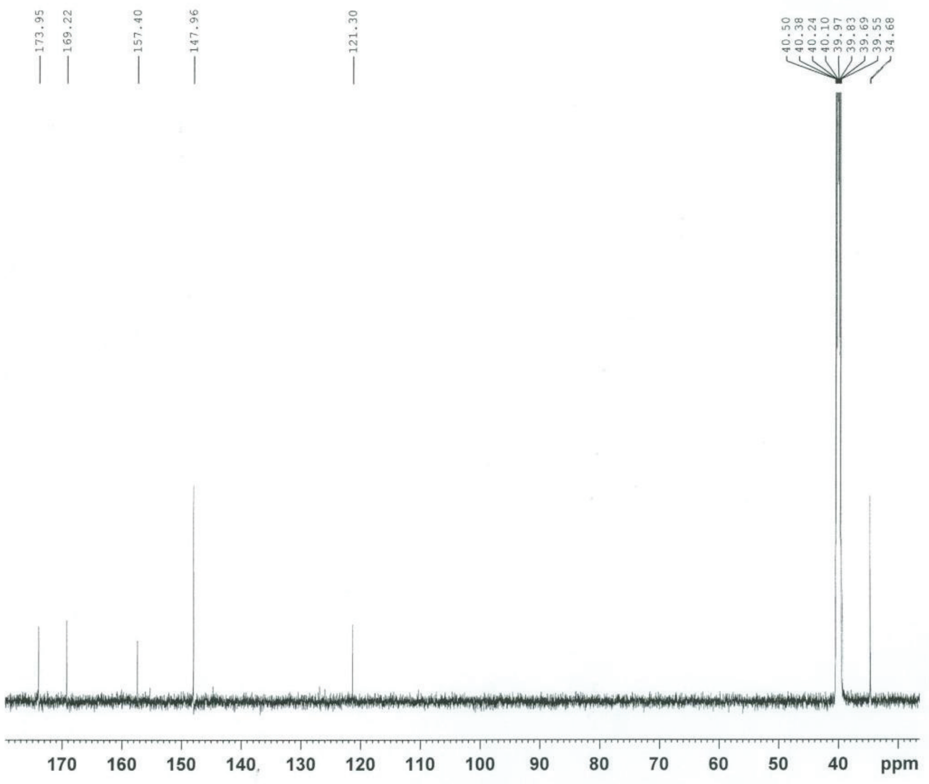

Compound **10e** ^{1}H NMR

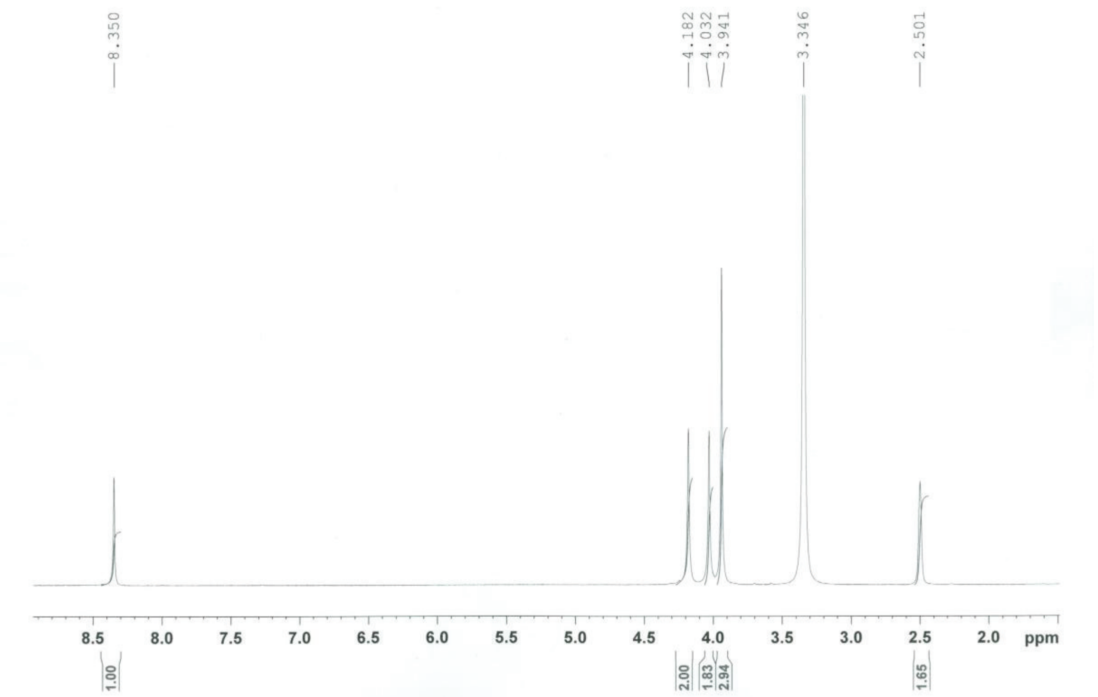

Compound **10e** ^{13}C NMR

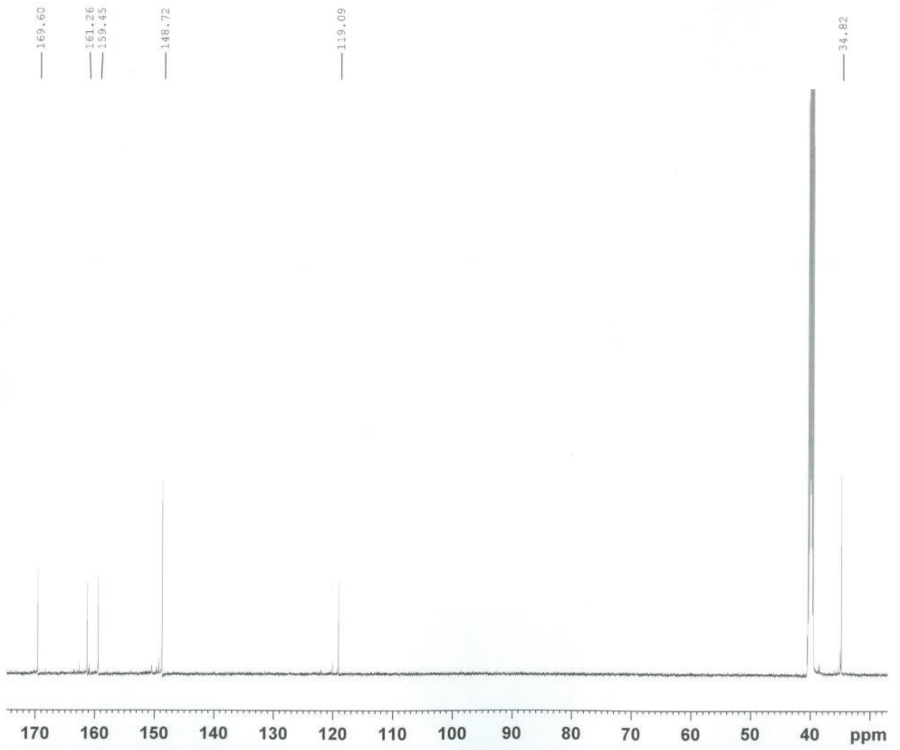

Compound **5a** HSQC

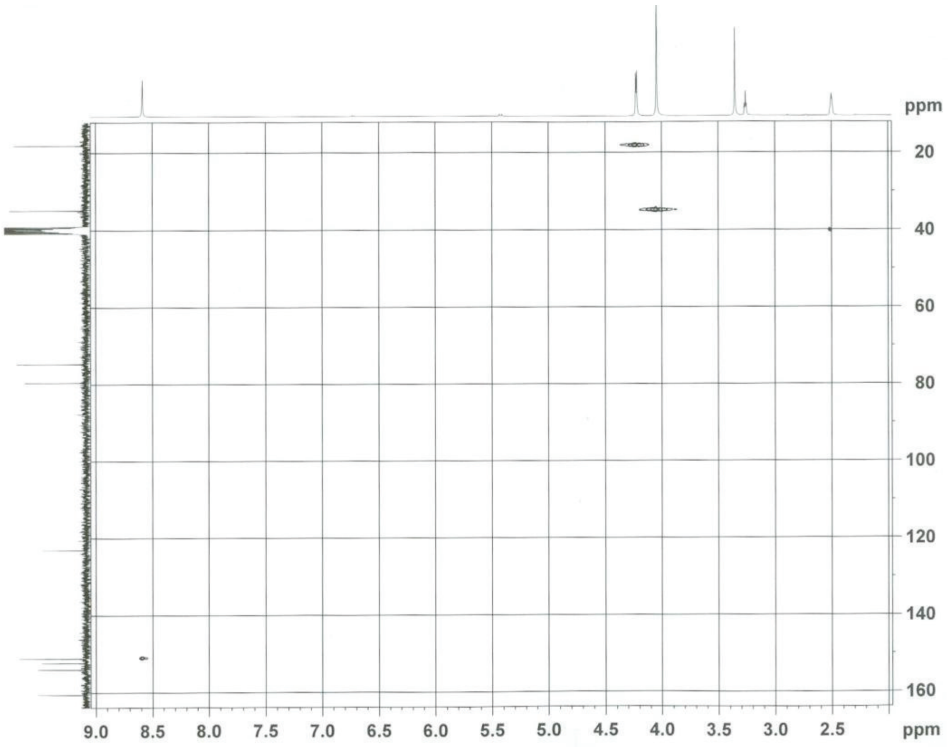

Compound **5a** HMBC

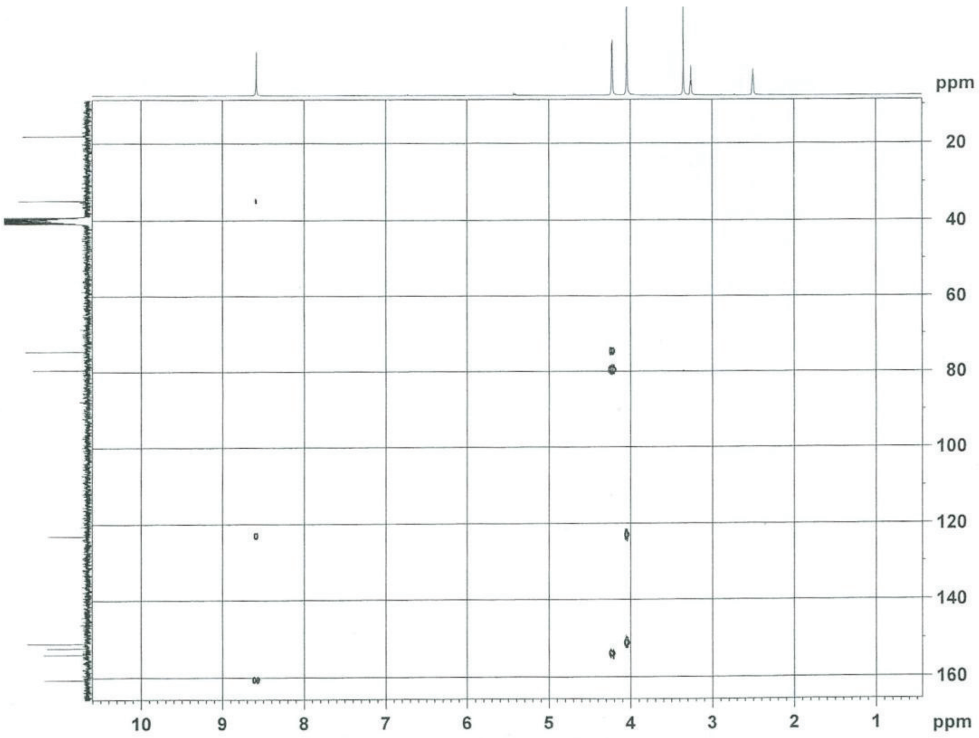

Compound **10e** HSQC

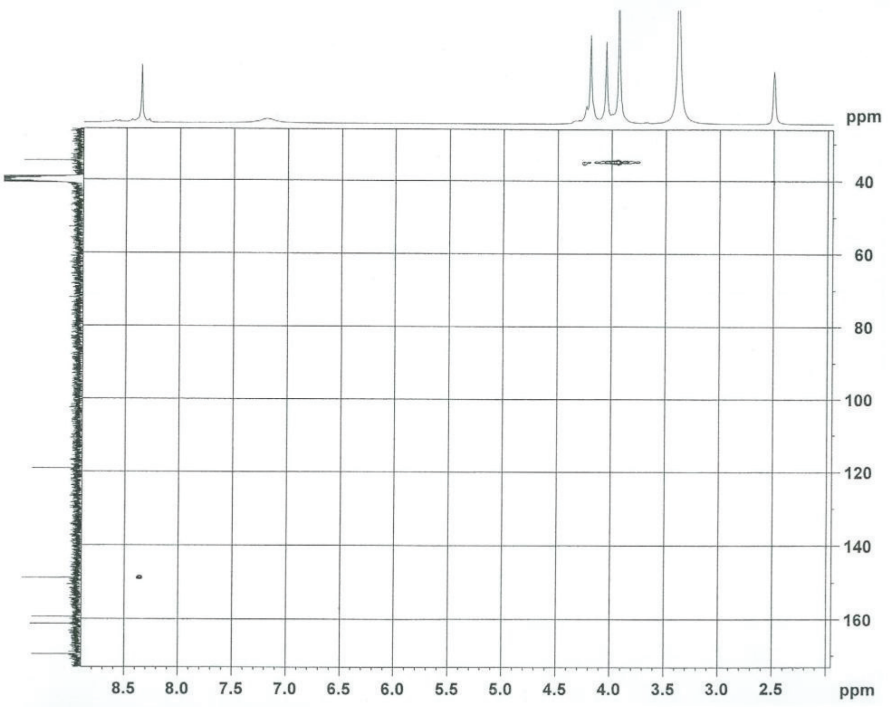

Compound **10e** HMBC

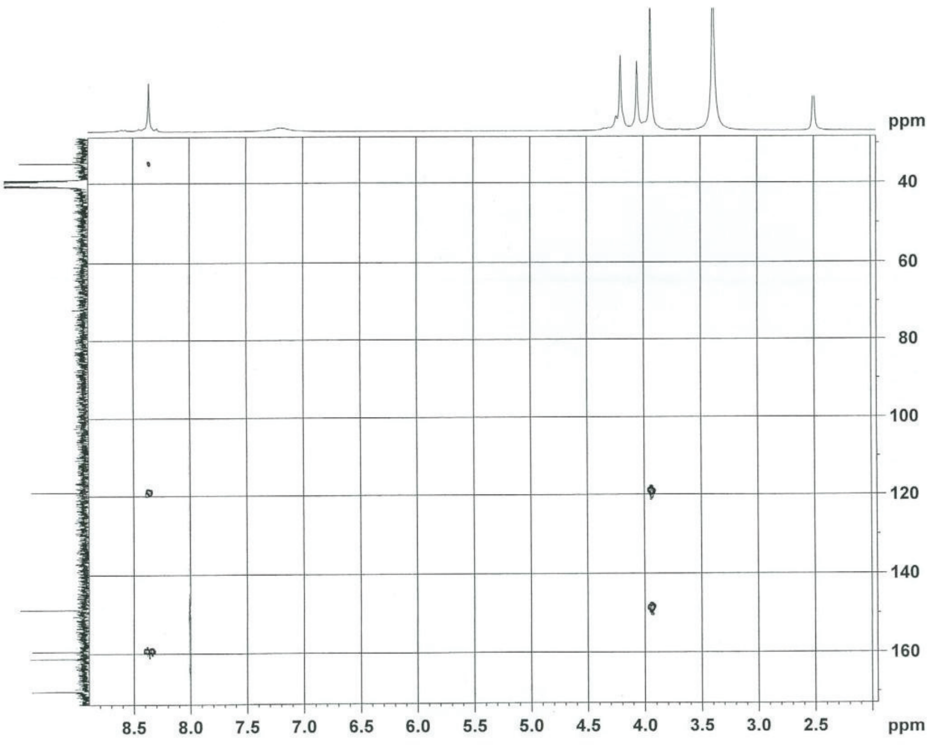

Supplement: Supplementary file 1 — Supplementary material 1 (PDF 5021 kb) [file 44_2015_1364_MOESM1_ESM.pdf]
